# Colistin-based combination therapy versus monotherapy for carbapenem-resistant gram-negative bacterial infections: a systematic review and meta-analysis

**DOI:** 10.3389/fcimb.2025.1729919

**Published:** 2026-01-12

**Authors:** Tingyu Yang, Hongjie Li, Xinqi Xu, Jiapan An, Zhongmou Zhang, Bin Li, Zhimin Dou

**Affiliations:** 1 The First School of Clinical Medicine, Lanzhou University, Lanzhou, China; 2 Department of Critical Care Medicine, The First Hospital of Lanzhou University, Lanzhou, China

**Keywords:** carbapenem-resistant gram-negative bacteria, colistin, mortality, combination therapy, monotherapy

## Abstract

**Objective:**

The objective of this study was to summarize available data on colistin (COL) combination therapy or monotherapy for carbapenem-resistant gram-negative bacteria (CR-GNB).

**Methods:**

Two reviewers independently evaluated and extracted data from PubMed, Embase, and Cochrane Library from inception to January 31, 2025, for studies comparing COL combination therapy with monotherapy in patients with CR-GNB infections. The primary outcome was all-cause mortality, and secondary outcomes included microbiological eradication rate, clinical improvement rate, length of stay (LOS), nephrotoxicity, and neurotoxicity. Differences for dichotomous outcomes were expressed as risk ratios (RRs) with 95% confidence intervals (CIs), whereas those for continuous outcomes were expressed as mean differences (MDs) with 95% confidence intervals (CIs). The risk of bias was assessed with the Cochrane tools. Certainty of evidence was assessed using GRADE. This systematic review was registered with PROSPERO (CRD42025636727).

**Results:**

A total of 26 eligible studies were included. Moderate-quality evidence indicates that compared with COL monotherapy, COL combination therapy significantly increased microbial eradication rate (RR 1.07, 95% CI 1.00–1.13, *p* = 0.04), particularly in infections caused by carbapenem-resistant *Acinetobacter baumannii* (CRAB). However, there were no significant differences in terms of 28-day all-cause mortality (RR 0.94, 95% CI 0.85–1.05, *p* = 0.30). In addition, low-quality evidence suggests that there were no significant differences were observed between COL monotherapy and COL combination therapy in terms of clinical improvement rate (RR 1.00, *p* = 0.97), intensive care units LOS (MD 0.67 days, *p* = 0.80), total LOS (MD 0.84 days, *p* = 0.67), nephrotoxicity (RR 0.98, *p* = 0.64) and neurotoxicity (RR 0.51, *p* = 0.14).

**Conclusion:**

Moderate-quality evidence suggests that COL combination therapy improved microbiological eradication rates in CRAB infections compared to monotherapy. However, high-quality RCTs are still needed to confirm the beneficial role of colistin-based combination therapy.

**Systematic review registration:**

https://www.crd.york.ac.uk/PROSPERO/myprospero, identifier CRD42025636727.

## 1 Introduction

In recent years, the widespread use of broad-spectrum antibiotics has led to a significant increase in the incidence of carbapenem-resistant gram-negative bacterial (CR-GNB) infections in intensive care units (ICU) (Qin et al., 2024). According to 2024 data from the China Antimicrobial Surveillance Network (CHINET), the carbapenem resistance rates are 82.5% for *Acinetobacter baumannii* (CRAB), 34.7% for *Klebsiella pneumoniae*, and 28.1% for *Pseudomonas aeruginosa* (China antimicrobial surveillance network (CHINET)). This resistance profile has severely limited clinical antibiotic options, contributing to an all-cause mortality rate of up to 40% in patients with CR-GNB infections, significantly worsening patient outcomes (Murray et al., 2022; Jean et al., 2022).

Colistin (COL), a polypeptide antibiotic, disrupts the outer membrane of CR-GNB and can enhance the bactericidal effects of other antibiotics (Tsuji et al., 2019). Owing to its sustained antibacterial activity against CR-GNB, COL is recognized as the definitive last-line therapeutic option for CR-GNB infections (Gales et al., 2011; Nang et al., 2021; Mousavi et al., 2025). However, COL’s narrow therapeutic window and dose-dependent nephrotoxicity limit its effectiveness as monotherapy, prompting the adoption of combination regimens in clinical practice (Kelesidis and Falagas, 2015; Karakonstantis et al., 2020).

Currently, the comparative efficacy of COL combination therapy versus monotherapy for CR-GNB infections remains controversial. Compared with monotherapy, observational studies suggest that COL combination therapy may improve clinical response rates and microbiological eradication rates and reduce patient mortality (Batirel et al., 2014; Abdelsalam et al., 2018; Hao et al., 2022). However, multiple meta-analyses have demonstrated no significant advantages of combination therapy in terms of mortality, microbiological clearance, or hospital length of stay (Gu et al., 2014; Cheng et al., 2018; Vardakas et al., 2018). Therefore, this study aims to incorporate the most recent clinical trials and provide an updated systematic assessment of the efficacy and safety differences between colistin monotherapy and combination regimens for CR-GNB infections.

## 2 Methods

The systematic review and meta-analysis were conducted in line with the Preferred Reporting Items for Systematic Reviews and Meta-Analyses (PRISMA) 2020 statement and registered their protocol in PROSPERO (CRD42025636727) (Page et al., 2021).

### 2.1 Search strategy

Two authors systematically searched the bibliographic databases, including PubMed, Embase, and the Cochrane Library, starting from their inception to January 31, 2025, with no limits for language and geographical region. The search strategies used a combination of the following search terms (1): carbapenem-resistant or carbapenemase-producing or carbapenem-nonsusceptible or multidrug-resistant gram-negative bacteria or extensively drug-resistant gram-negative bacilli; (2) Colistin or Colimycin or Colisticin or Polymyxin E or Colistin Sulfate or Sulfate or Colistin or Totazina or Coly Mycin. The search strategies are depicted in **Supplementary Table S1.**

### 2.2 Inclusion and exclusion criteria

The inclusion criteria were as follows: (1) study designs limited to observational studies or randomized controlled trials (RCTs); (2) participants with microbiologically confirmed CR-GNB infections; (3) intervention measures included COL combination therapy and monotherapy with intravenous administration; and (4) reported one of the following endpoints: 28-day all-cause mortality, in-hospital all-cause mortality, clinical improvement rate, microbiological eradication, length of stay (LOS) in the ICU, total LOS, nephrotoxicity and neurotoxicity. No exclusion criteria were set for the dose of COL.

The criteria for exclusion were as follows: (1) animal experiments, *in vitro* studies, pediatric research, editorial letters, comments, guidelines, conference abstracts, systematic reviews, meta-analyses; (2) studies with incomplete outcome data or noncomparable outcome metrics; and (3) studies enrolling fewer than 10 patients.

### 2.3 Literature screening and data extraction

Two authors independently assessed the relevant studies according to inclusion/exclusion criteria, and negotiated with a third party to resolve any disagreements. The study data were independently extracted by two reviewers in a standardized established data format, including the following study characteristics: first author’s name, type of study design, publication year, country, sex and age of patients, Acute Physiology and Chronic Health Evaluation (APACHE II) score and Sequential Organ Failure Assessment (SOFA), sample size, type of pathogen, co-administration of other antibiotics, 28-day all-cause mortality, in-hospital all-cause mortality, microbiological eradication rate, clinical improvement rate, length of stay (LOS), nephrotoxicity, and neurotoxicity. Any disagreements were resolved through discussion and consultation. The reviewers attempted to establish contact with the authors via email in cases where insufficient data were available.

### 2.4 Definitions

CR-GNB are referred to as gram-negative bacteria (GNB) identified from clinical specimen cultures that demonstrate resistance to imipenem, meropenem, and ertapenem, as indicated by antimicrobial susceptibility testing results. The primary outcome was 28-day all-cause mortality, while secondary outcomes included in-hospital all-cause mortality, clinical improvement rate, microbiological eradication rate, incidence of nephrotoxicity and neurotoxicity, ICU length of stay (LOS), and total hospital LOS. The all-cause mortality referred to the all-cause hospital mortality. Clinical improvement was defined as the resolution of infection-related signs or symptoms without recurrence or survival during the follow-up period. Microbiological eradication was defined as the absence of baseline pathogens in cultures obtained during follow-up. Nephrotoxicity was defined as a marked increase in serum creatinine level or an obvious decrease in glomerular filtration rate, which prompted renal replacement therapy and was diagnosed based on the classification of risk, injury, failure, loss, and end-stage kidney disease criteria (RIFLE) (Hartzell et al., 2009). Neurotoxicity is defined as the occurrence of symptoms such as dizziness, muscle weakness, facial and peripheral paraesthesia, visual disturbances, vertigo, confusion, hallucinations, seizures, ataxia, partial deafness, and neuromuscular blockade during the administration of polymyxins (Molina et al., 2009). The critically ill patient is defined as having an Acute Physiology and Chronic Health Evaluation II score greater than 15. To distinguish publications from the same first author published in the same year, alphabetical suffixes (e.g., “Author2025a”, “Author2025b”) were added.

### 2.5 Risk of bias assessment

Quality assessment was performed independently by two investigators, using the ROBINS-I for observational studies and the ROB 2.0 for RCTs (Sterne et al., 2016; Sterne et al., 2019). The classification of the overall risk of bias for each included study was as follows: low, if there was low risk of bias in all domains, unclear, if there was unclear risk of bias in one or more domains without any judgment of high risk of bias, and high, if there was high risk of bias in one or more domains (Alhazzani et al., 2018). Discrepancies were resolved by a third investigator after a joint re-evaluation of the original studies was conducted by the previous reviewers.

### 2.6 Quality of evidence

Outcomes were rated according to the Grading of Recommendations, Assessment, Development, and Evaluations (GRADE) Framework (Guyatt et al., 2008). Certainty of evidence could be considered as ‘very low’, ‘low’, ‘moderate’, or ‘high’ depending on the number of downgrades attributed to each of the five topics: (1) risk of bias, (2) imprecision, (3) inconsistency, (4) indirectness, and (5) publication bias. *Risk of bias* was rated based on the outline in the section ‘Risk of bias assessment’. *Impression* was deemed to be present if outcomes were calculated from only a few studies with small sample sizes, or if decision-making would differ when the lower and upper confidence limits were considered as the real effect. *Publication bias* was determined by assessing funnel plots. *Indirectness* was deemed if the study did not use a placebo or control as a comparator, whereas *inconsistency* was determined according to the heterogeneity measures (I*^2^* or tau^2^) (Guyatt et al., 2008).

### 2.7 Statistical analysis

All statistical analyses were conducted using a random-effects model via Review Manager (RevMan) version 5.4 (The Nordic Cochrane Centre, Copenhagen, Denmark). Differences were expressed as odds ratios (ORs) with 95% confidence intervals (CIs) for dichotomous outcomes and as mean differences (MDs) with 95% CIs for continuous outcomes. Heterogeneity was assessed using the inconsistency index (I2) and the Q statistic. A P-value of less than 0.10 for the Q statistic was considered significant (Higgins and Thompson, 2002). To evaluate the impact of potential outlier studies on the stability of effect estimates, sensitivity analyses were conducted using the leave-one-out method (Deeks et al., 2024). Subgroup analyses were conducted for specific categories, including study design (RCTs and observational studies), study setting (multicenter vs. single-center), pathogen subtype (CRAB-infected patients only), antibiotic regimen (COL + meropenem, COL+ tigecycline, COL + rifampicin), and baseline severity (critically ill and stable patients). Publication bias was assessed using funnel plots and Egger’s test, conducted with R version 4.4.2 (R Foundation for Statistical Computing, Vienna, Austria). The presence of publication bias was inferred when both indicators yielded statistically significant results. A two-tailed *P*-value less than 0.05 was considered statistically significant (Page MJ and Sterne, 2024).

## 3 Results

### 3.1 Study selection

**Figure 1:**
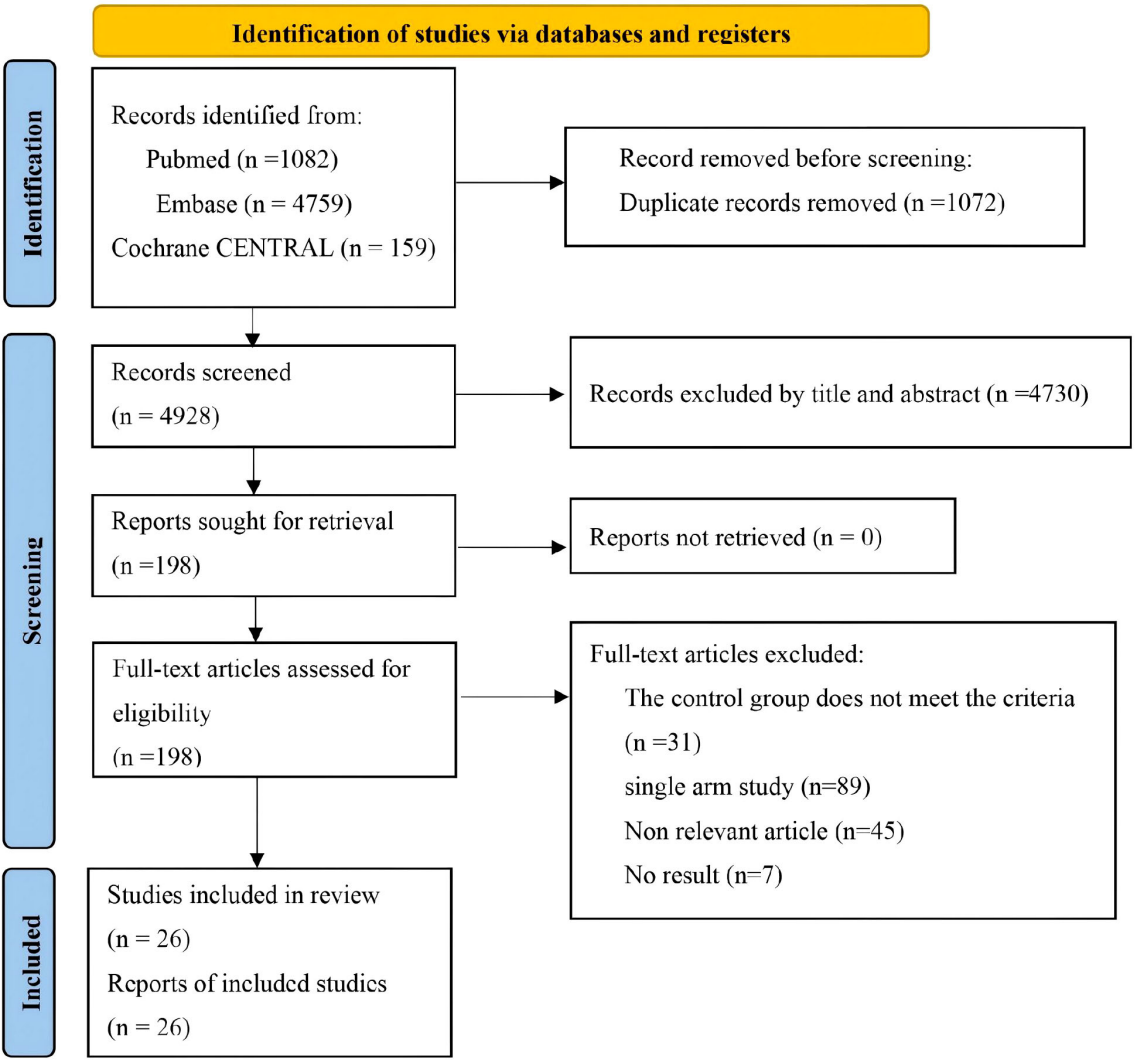
PRISMA flowchart of study selection.

Systematic searches across PubMed (n=1082), Embase (n=4759), and the Cochrane Library (n=159) initially identified 6000 records, with 4928 studies retained for screening after duplicate removal. A total of 4782 publications unrelated to the research topic were excluded by screening the titles and abstracts. Subsequently, the full text of the remained 146 articles was thoroughly examined, and 120 were excluded for not meeting the inclusion criteria. Finally, 26 studies were included in the analysis (Falagas et al., 2006; Aydemir et al., 2013; Durante-Mangoni et al., 2013; Batirel et al., 2014; Kalin et al., 2014; Sirijatuphat and Thamlikitkul, 2014; Rigatto et al., 2015; Yilmaz et al., 2015; Ghafur et al., 2016; Parchem et al., 2016; Ghafur et al., 2017; Abdelsalam et al., 2018; Amat et al., 2018; Makris et al., 2018; Paul et al., 2018; Park et al., 2019; Shi et al., 2019; Katip and Uitrakul, 2020; Katip et al., 2020; Park et al., 2020; Katip and Oberdorfer, 2021; Chang et al., 2022; Hao et al., 2022; Sirijatuphat et al., 2022; Kaye et al., 2023; Katip et al., 2024). The analysis included a total of 3964 patients (2135 received COL combination therapy and 1829 received COL monotherapy). **Figure 1** presents the literature selection flowchart. **Table 1** and **Table 2** summarize the baseline characteristics of the included RCTs and observational studies, respectively.

**Table 1:** Characteristics of the included RCTs.

References	Study setting	Study population	Country	No. of patients (n)		Combine regimen	Age	Female, n (%)	Patients with bacteremia, n (%)	Outcome of interest
				Combine	Mono					
Abdelsalam MFA et al. (2018)	multicenter	CRE (*K. pneumonia*)	Egypt	30	30	MEM	NR	32 (53)	28(47)	In-hospital mortality, AEs
Aydemir H et al. (2013)	single center	CRAB	Turkey	21	22	RFP	61 ± 20	13 (31.2)	8 (18.6)	Clinical cure/improvement rates, micro. eradication rates, mortality, VAP-related mortality
Makris D et al. (2018)	multicenter	MDR-AB	Greece	20	19	Amp-SB	NR	12(30.8)	18(46.2)	Mortality, micro. eradication rates, AEs, clinical cure/improvement rates
Sirijatuphat R et al. (2014)	open-label, single center	CRAB	Thailand	47	47	FFM	NR	50(53.2)	10(10.6)	Mortality, clinical cure/improvement rates, micro. eradication rates, total hospital LOS, AEs
Sirijatuphat R et al. (2022)	open-label, single center	CRAB	Thailand	28	28	SIT	69.2 ± 12.2	22 (39.3)	12 (21.4)	Clinical cure/improvement rates, micro. eradication rates, total hospital LOS, mortality, AEs
Paul M et al. (2018)	open-label, parallel, multicenter	CRAB (76.85%), CRE (18%). CRPA,	Israel, Greece, Italy,	208	198	MEM	NR	255(62.8)	173(42.6)	Clinical cure/improvement rates, mortality, micro. eradication rates, AEs
Durante-Mangoni E et al. (2013)	open-label, parallel, multicenter	XDR-AB	Italian	104	105	RFP	62 ± 15.4	72(34.4)	42 (20.1)	Mortality, micro. eradication rates, total hospital LOS, AEs
Kaye KS et al. (2023)	double-blind, placebo-controlled trial, multicenter	XDR-AB (77.78%), XDR- CRPA (10.17%), CRE (16.31%).	USA, Thailand, Taiwan, Israel, Greece, Italy, and Bulgaria	210	213	MEM	NR	158(37.4)	148(35)	ICU LOS, mortality, clinical cure/improvement rates, micro. eradication rates, AEs

MEM, meropenem; RFP, rifampicin; FFM, fosfomycin; Amp-SB, ampicillin-sulbactam; SIT, sitafloxacin; MINO, minocycline; VAP, ventilator-associated pneumonia; XDR-AB, extremely drug-resistant *Acinetobacter baumannii*; CRAB, carbapenem-resistant *Acinetobacter baumannii*; CRE, carbapenem-producing *enterobacterales*; CRPA, *carbapenem-resistant pseudomonas aeruginosa*; AE, adverse event; NR, no report; USA, united states of America; LOS, length of stay; combine, combine therapy; mono, therapy.

**Table 2:** Characteristics of the included observational studies.

References	Study setting	Study population	Country	No. of patients (n)		Combine regimen	Age	Female, n (%)	Patients with bacteremia, n (%)	Outcome of interest
				Combine	Mono					
Amat T et al. (2018)	Ret. multicenter	CRAB	Spanish	42	76	TGC	57 ± 15	44(37.3)	118 (100)	Mortality
Batirel A et al. (2014)	Ret. multicenter	XDR-AB	Turkey	214	36	CB, SB, TGC, AMK, NTM, GEN, AGs, RFP, PTZ	NR	88(35.2)	250(100)	Mortality, in-hospital mortality, micro. eradication rates, clinical cure/improvement rates, AEs
Kalin G et al. (2014)	Ret. single center	MDR-AB	Turkey	37	52	SB	NR	35(39.3)	NR	Clinical cure/improvement rates, micro. eradication rates, mortality, ICU LOS, AEs
Katip W et al. (2020)	Ret. single center	CRAB	Thailand	131	193	MEM	NR	127(39.2)	115(35.5)	Total hospital LOS, clinical cure/improvement rates, micro. eradication rates, AEs, and mortality
Katip W et al. (2020)	Ret. single center	CRAB	Thailand	124	124	MEM	NR	158(63.7)	2(0.8)	Total hospital LOS, mortality, clinical cure/improvement rates, micro. eradication rates, AEs
^a^ Chang K et al. (2022)	Ret. multicenter	CRAB (50.79%), CRPA (14.66%), CRKP (43.36%)	China	92	99	TGC	NR	43(22.5)	NR	Total hospital LOS, clinical cure/improvement rates, mortality
Falagas ME et al. (2006)	Ret. single center	CRAB (49.33%), CRPA (33.33%), CRKP (12%), SMA, *E. coli*, EC	Greece	57	14	MEM	NR	29(40.8)	17(23.9)	Total hospital LOS, clinical cure/improvement rates, AEs, mortality
Ghafur A et al. (2016)	Ret. single center	CRE (60.44%): *E. coli*, CRPA, CRKP, CRAB.	India	65	26	MEM, and /or TGC,	NR	33(36.3)	NR	Mortality
Ghafur A et al. (2017	Ret. single center	CRAB (35%), CRPA (15%), CRKP (42%), *E. coli* (5%).	India	92	61	TGC, CB, SB	NR	35(22.9)	NR	Mortality, clinical cure/improvement rates, micro. eradication rates
Hao M et al. (2022)	Ret. multicenter	CRAB (42.5%), CRPA (25%), CRKP(21.25%), SMA, and CF.	China	34	26	TGC, CB, CPZ/SB, PTZ, CAZ-AVI, AGs, LVFX.	NR	21(26.3)	12(15)	Clinical cure/improvement rates, AEs, micro. eradication rates, total hospital LOS, mortality
Katip W et al. (2021)	Ret. single center	CRAB	Thailand	115	115	VAN	NR	154(67)	1(0.4)	Total hospital LOS, mortality, clinical cure/improvement rates, micro. eradication rates, AEs
Katip W et al. (2024)	Ret. single center	CRE	Thailand	153	67	FFM	NR	89(40.5)	16(7.3)	Mortality, clinical cure/improvement rates, micro. eradication rates
Park SY et al. (2019)	Ret. single center	CRAB,	Korea	31	40	MEM	NR	29(40.8)	71(100)	Mortality, clinical cure/improvement rates
Shi H et al. (2019)	Ret. single center	CRAB	Korea	83	77	CB	NR	46(28.8)	NR	Mortality, clinical cure/improvement rates, micro. eradication rates
Rigatto MH et al. (2015)	Ret. multicenter	XDR-AB (82.2%), XDR-PA (17.8%).	Brazil.	33	68	MEM, IPM, Amp-SB, PTZ, RFP, AMK,	NR	45(44.6)	19 (18.8)	Mortality
Parchem NL et al. (2016)	Ret. single center	CRAB (82.2%), CRPA (20%), CRKP (1.1%).	USA	41	49	TGC, MINO, Amp-SB, IPM/CS, DPZ	NR	38(42.2)	NR	LOS, ICU LOS, mortality
Park JJ et al. (2020)	Ret. single center	CRAB (58.33%), CRPA (36.90%), CRKP (4.76%),	Korea	52	32	CB, TGC	NR	14(17.5)	NR	Micro. eradication rates, ICU LOS, total hospital LOS, mortality, AEs
Yilmaz GR et al. (2015)	Ret. single center	MDR/XDR-AB	Turkey	53	17	SB, CB	NR	37(52.9)	NR	Clinical cure/improvement rates, micro. eradication rates, total hospital LOS, mortality, AEs

MEM, meropenem; IPM, imipenem; TGC, tigecycline; RFP, rifampicin; CB, carbapenem; CPZ/SB, cefoperazone/sulbactam; AMK, amikacin; NTM, netilemic; GEN, gentamicin; AGs, aminoglycoside; PTZ, piperacillin/tazobactam; CAZ-AVI, ceftazidime/avibactam; LVFX, levofloxacin; VAN, vancomycin; FFM, fosfomycin; Amp-SB, ampicillin-sulbactam; SIT, sitafloxacin; MINO, minocycline; IPM/CS, imipenem/cilastatin; XDR-AB, extremely drug-resistant *Acinetobacter baumannii*; CRAB, carbapenem-resistant *Acinetobacter baumannii*; CRE, carbapenem-producing *enterobacterales*; CRPA, *carbapenem-resistant pseudomonas aeruginosa*; CRKP, carbapenem-resistant *Klebsiella Pneumoniae*, SMA, *Stenotrophomonas maltophilia*; EC, *enterobacter cloacae*; E. coli, *escherichia coli*; CF, *Citrobacter freudii*; AE, adverse event; NR, no report; USA, united states of America; LOS, length of stay; Ret, retrospective; combine, combine therapy; mono, therapy.

a Two groups from the three study groups that met the research requirements were compared.

### 3.2 Quality assessment

**Figure 2:**
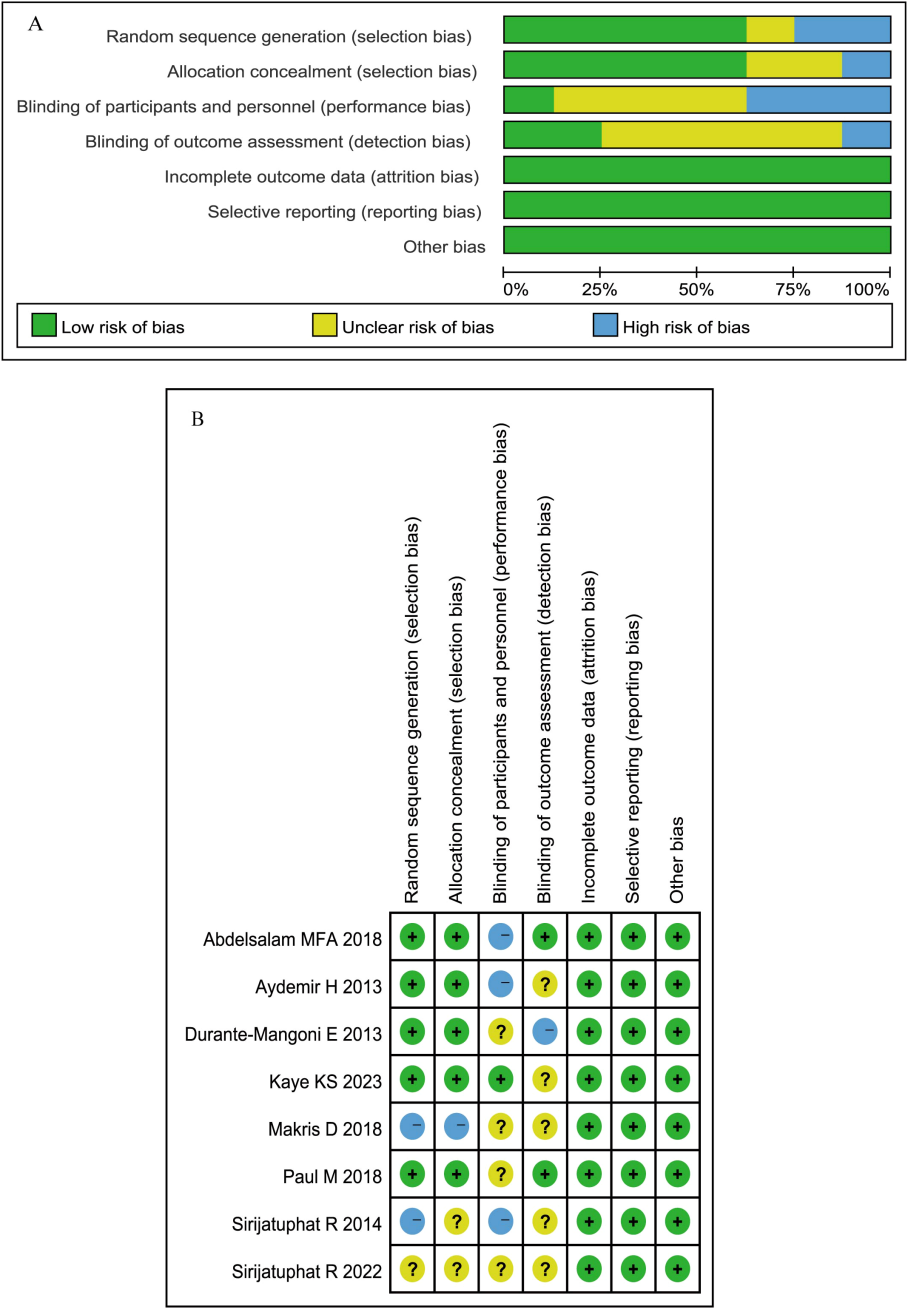
**(A)** Quality assessment summary of the RCTs. **(B)** Quality assessment details of the RCTs.

**Figure 3:**
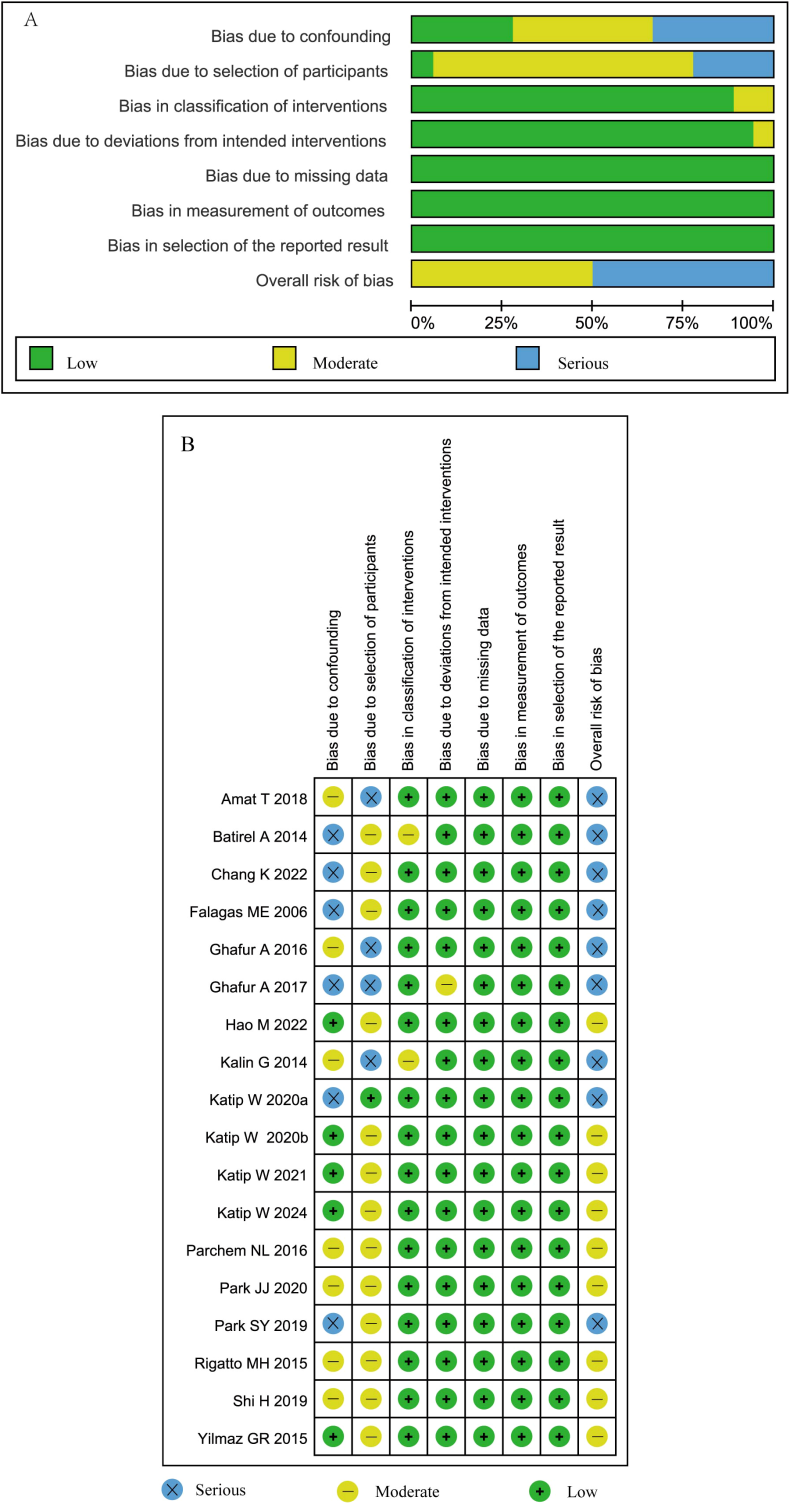
**(A)** Quality assessment summary of observational studies. **(B)** Quality assessment details of the observational studies. Katip W2020a and Katip W2020b denote two independent studies published by the Katip W research team in 2020.

The risk of bias assessment for the 8 RCTs via the ROB 2.0 tool (**Figure 2**) revealed high risk in 5 studies and moderate risk in 3 studies, with primary biases stemming from deficiencies in double-blinding implementation and inadequate allocation concealment. For the 18 observational studies evaluated with the ROBINS-I tool (**Figure 3**), 9 studies demonstrated a moderate risk of bias and 9 exhibited a serious risk of bias, predominantly attributed to uncontrolled confounding factors, heterogeneity in intervention protocols, and selection bias.

### 3.3 Characteristics of the included studies

**Table 1** shows the main characteristics of the included studies. All 11 included studies were conducted from 2006 to 2024, with sample sizes ranging from 14 to 214 participants. Eight studies were RCTs (Aydemir et al., 2013; Durante-Mangoni et al., 2013; Sirijatuphat and Thamlikitkul, 2014; Abdelsalam et al., 2018; Makris et al., 2018; Paul et al., 2018; Sirijatuphat et al., 2022; Kaye et al., 2023), while 18 studies were observational (Falagas et al., 2006; Batirel et al., 2014; Kalin et al., 2014; Rigatto et al., 2015; Yilmaz et al., 2015; Ghafur et al., 2016; Parchem et al., 2016; Ghafur et al., 2017; Amat et al., 2018; Park et al., 2019; Shi et al., 2019; Katip and Uitrakul, 2020; Katip et al., 2020; Park et al., 2020; Katip and Oberdorfer, 2021; Chang et al., 2022; Hao et al., 2022; Katip et al., 2024).Seven studies were multicenter (Durante-Mangoni et al., 2013; Rigatto et al., 2015; Abdelsalam et al., 2018; Makris et al., 2018; Paul et al., 2018; Chang et al., 2022; Hao et al., 2022; Kaye et al., 2023) while the others were single-center (Falagas et al., 2006; Aydemir et al., 2013; Kalin et al., 2014; Sirijatuphat and Thamlikitkul, 2014; Yilmaz et al., 2015; Ghafur et al., 2016; Parchem et al., 2016; Ghafur et al., 2017; Park et al., 2019; Shi et al., 2019; Katip and Uitrakul, 2020; Katip et al., 2020; Park et al., 2020; Katip and Oberdorfer, 2021; Sirijatuphat et al., 2022; Katip et al., 2024). The study population had a mean age of 61.4 ± 15.8 years, with a male predominance (57.6%). The research spanned multiple countries, such as China, the United States, Greece, Italy, Turkey, Thailand, South Korea, Brazil, and India. Fourteen studies reported exclusively on CRAB infections. Two studies focused solely on carbapenem-resistant Enterobacteriaceae (CRE). Common infection sites include the respiratory tract, bloodstream, abdominal cavity, and urinary tract. The combination regimens encompassed carbapenems (e.g., meropenem, imipenem), aminoglycosides (e.g., amikacin, gentamicin), β-lactam/β-lactamase inhibitor combinations (e.g., piperacillin/tazobactam, ceftazidime/avibactam, cefoperazone/sulbactam), fluoroquinolones (e.g., levofloxacin, sitafloxacin), and other antibiotics (e.g., tigecycline, rifampicin). Notably, seven studies utilized triple or higher-order COL combination protocols (e.g., COL plus meropenem plus tigecycline). Baseline data indicated greater disease severity and Charlson Comorbidity Index (CCI) scores in the combination therapy group than in the monotherapy group. The detailed distributions of infection sites, disease severity, and the CCI are provided in the supplementary file (**Supplementary file, Table S2**).

### 3.4 Primary outcomes

**Figure 4:**
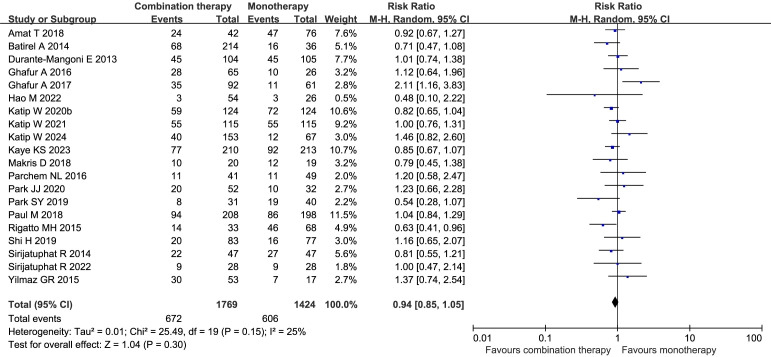
Forest plot of 28-day all-cause mortality rates between COL combination therapy and monotherapy.

**Figure 5:**
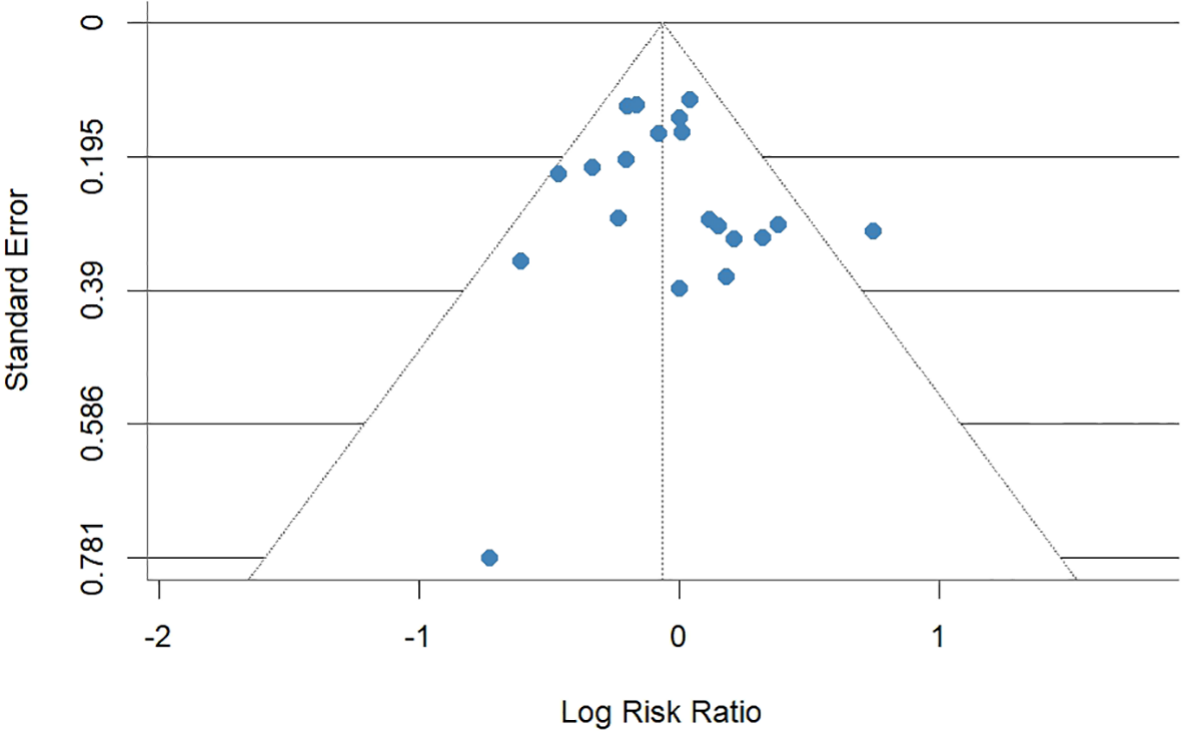
Funnel plot of 28-day all-cause mortality rates between COL combination therapy and monotherapy.

All twenty studies (n=3193) were accessible to compare the 28-day all-cause mortality rate (Durante-Mangoni et al., 2013; Batirel et al., 2014; Sirijatuphat and Thamlikitkul, 2014; Rigatto et al., 2015; Yilmaz et al., 2015; Ghafur et al., 2016; Parchem et al., 2016; Ghafur et al., 2017; Amat et al., 2018; Makris et al., 2018; Paul et al., 2018; Park et al., 2019; Shi et al., 2019; Katip and Uitrakul, 2020; Park et al., 2020; Katip and Oberdorfer, 2021; Hao et al., 2022; Sirijatuphat et al., 2022; Kaye et al., 2023; Katip et al., 2024). The 28-day all-cause mortality rate was 38% in the COL combination therapy group and 42.6% in the COL monotherapy group. There was no significant difference between patients treated with colistin and other antibiotics (RR 0.94, 95% CI 0.85–1.05, *I²* = 25%, *p* = 0.30) (**Figure 4**). The combination therapies included dual therapies and a small number of triple or quadruple therapies. **Figure 5** shows a funnel plot of the 28-day all-cause mortality rate, which shows no evidence of publication bias. In addition, the results of Egger’s test indicated a low risk of publication bias (p = 0.5156). Sensitivity analysis via the leave-one-out method confirmed the robustness of the findings. Subgroup analyses stratified by study design, study setting, pathogen subtype (CRAB or CRE), antibiotic regimen, and baseline severity were conducted. The results revealed no significant difference in 28-day all-cause mortality between the two groups across all subgroups (**Table 3**) (**Supplementary file, Figure S1**).

**Table 3:** Subgroup analysis of 28-day all-cause mortality between COL combination therapy and monotherapy.

	Number of studies	Number of patients	Risk ratio	95%CI	I^2^
Study design					
RCT	6	1227	0.93	[0.82, 1.06]	0%
Retrospective observational study	14	1966	0.97	[0.82, 1.15]	43%
Study setting					
Single center	12	1567	1.04	[0.88, 1.25]	35%
Multicenter	8	1626	0.89	[0.79, 1.00]	3%
Pathogen subtype					
CRAB	11	1545	0.90	[0.80, 1.01]	0%
Antibiotic regimen					
COL+MEM	6	1239	0.86	[0.74, 1.00]	27%
COL+TGC	2	163	0.96	[0.71, 1.29]	0%
COL+RFP	2	278	1.02	[0.76, 1.37]	0%
Disease severity					
Critically ill patients	5	816	0.90	[0.76, 1.05]	0%
Stable patients	2	450	1.11	[0.79, 1.56]	30%

COL, colistin; CI, confidence intervals; RCT, randomized controlled trials; CRAB, carbapenem-resistant Acinetobacter baumannii; MEM, meropenem; TGC, tigecycline; RFP, rifampicin.

### 3.5 Secondary outcomes

#### 3.5.1 In-hospital mortality

**Figure 6:**
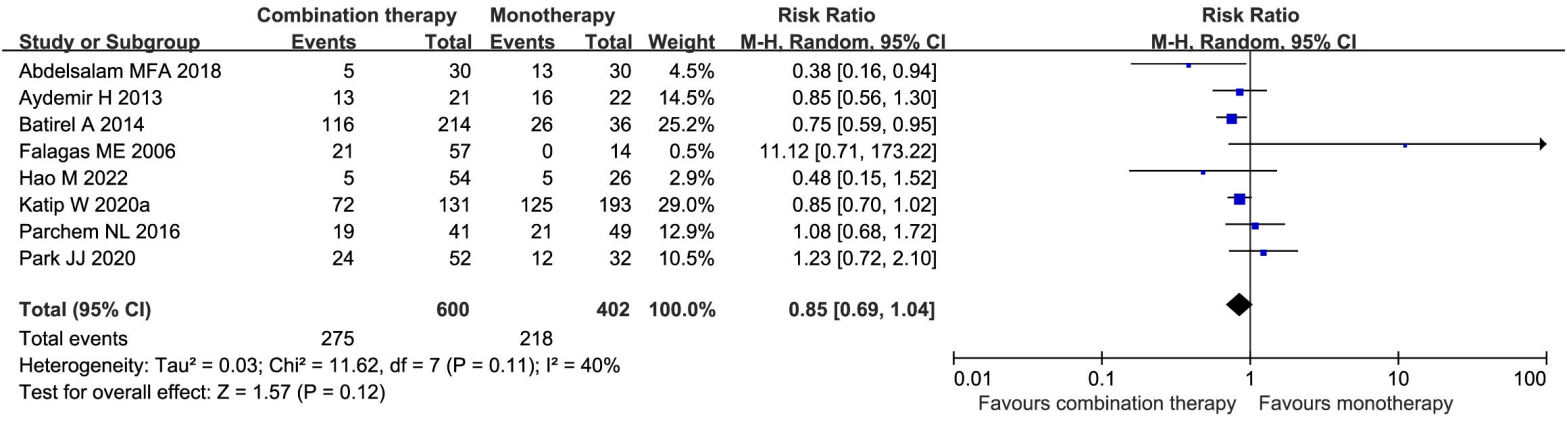
Forest plot of in-hospital mortality rates between COL combination therapy and monotherapy.

Eight studies (Falagas et al., 2006; Aydemir et al., 2013; Batirel et al., 2014; Parchem et al., 2016; Abdelsalam et al., 2018; Katip et al., 2020; Park et al., 2020; Hao et al., 2022), including 1002 patients, reported the in-hospital mortality in the COL combination therapy group compared with the COL monotherapy group, but it was not statistically significant (RR 0.85, 95% CI 0.69–1.04, *I²* = 40%, *p* = 0.12) (**Figure 6**). The results of Egger’s test indicated a low risk of publication bias (*p* = 0.6635). The subgroup analyses of observational studies and the RCTs revealed no difference (**Supplementary file, Figure S2**).

#### 3.5.2 LOS

**Figure 7:**
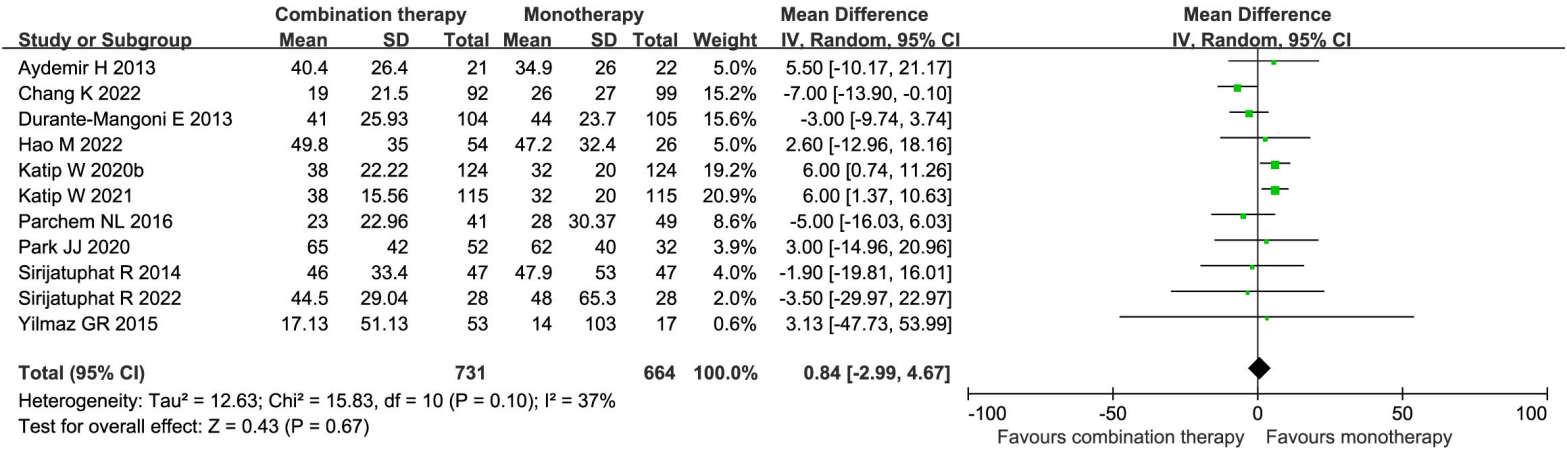
Forest plot of total LOS (days) between COL combination therapy and monotherapy.

**Figure 8:**
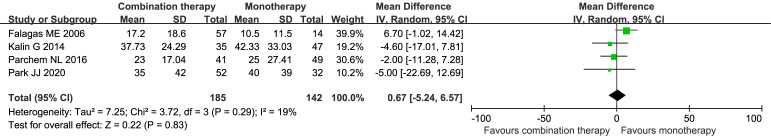
Forest plot of ICU LOS (days) between COL combination therapy and monotherapy.

Eleven studies (1395 patients)reported the total hospital LOS in the COL combination therapy compared with the monotherapy (Aydemir et al., 2013; Durante-Mangoni et al., 2013; Sirijatuphat and Thamlikitkul, 2014; Yilmaz et al., 2015; Parchem et al., 2016; Katip and Uitrakul, 2020; Park et al., 2020; Katip and Oberdorfer, 2021; Chang et al., 2022; Hao et al., 2022; Sirijatuphat et al., 2022), but there was no statistically significant difference (MD 0.84 days, 95% CI -2.99–4.67, *I²* = 37%, *p* = 0.67) (**Figure 7**). The risk of publication bias was low, as shown by Egger’s test (*p* = 0.7719). Subgroup analyses of observational studies and the RCTs indicated no significant differences between the groups (**Supplementary file, Figure S3**). The four studies (327 patients) also showed no significant difference in the ICU LOS between the two groups (MD 0.67 days, 95% CI -5.24–6.57, *I²* = 19%, *p* = 0.83) (Falagas et al., 2006; Kalin et al., 2014; Parchem et al., 2016; Park et al., 2020) (**Figure 8**). Egger’s test revealed a low risk of publication bias (*p* = 0.1382).

#### 3.5.3 Clinical improvement rate

**Figure 9:**
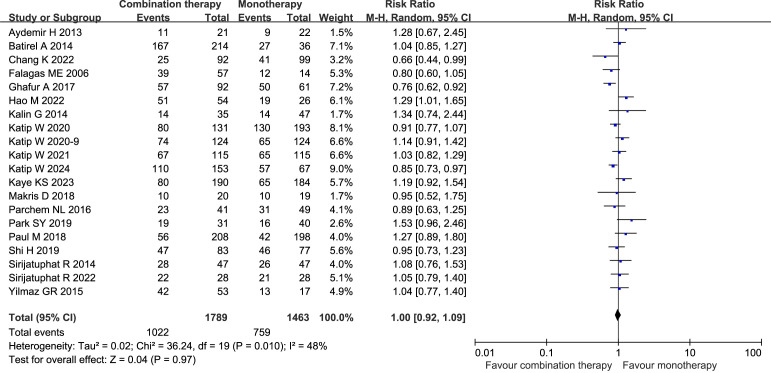
Forest plot of clinical improvement rates between COL combination therapy and monotherapy. Katip W2020a and Katip W2020b denote two independent studies published by the Katip W research team in 2020.

Twenty studies (3252 patients) reported no statistical difference in the clinical improvement rate (RR 1.00, 95% CI 0.92–1.09, *I²* = 48%, *p* = 0.97) (**Figure 9**) (Falagas et al., 2006; Aydemir et al., 2013; Batirel et al., 2014; Kalin et al., 2014; Sirijatuphat and Thamlikitkul, 2014; Yilmaz et al., 2015; Parchem et al., 2016; Ghafur et al., 2017; Makris et al., 2018; Paul et al., 2018; Park et al., 2019; Shi et al., 2019; Katip and Uitrakul, 2020; Katip et al., 2020; Katip and Oberdorfer, 2021; Chang et al., 2022; Hao et al., 2022; Sirijatuphat et al., 2022; Kaye et al., 2023; Katip et al., 2024). Additionally, the results of Egger’s test showed a low risk of publication bias (*p* = 0.0896). Subgroup analysis of the observational studies, the RCTs, single center study, multicenter study, CRAB, COL with meropenem, critically ill patients, and stable patients showed no significant differences between the groups (**Supplementary file, Figure S4**).

#### 3.5.4 Microbiological eradication rate

**Figure 10:**
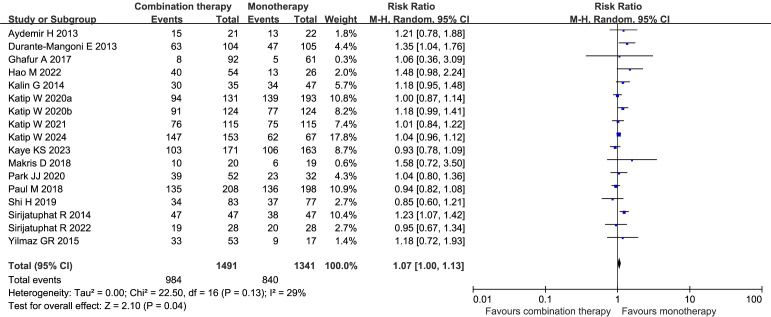
Forest plot of microbiological eradication rates between COL combination therapy and monotherapy. Katip W2020a and Katip W2020b denote two independent studies published by the Katip W research team in 2020.

Seventeen studies (Aydemir et al., 2013; Durante-Mangoni et al., 2013; Kalin et al., 2014; Sirijatuphat and Thamlikitkul, 2014; Yilmaz et al., 2015; Ghafur et al., 2017; Makris et al., 2018; Paul et al., 2018; Shi et al., 2019; Katip and Uitrakul, 2020; Katip et al., 2020; Park et al., 2020; Katip and Oberdorfer, 2021; Hao et al., 2022; Sirijatuphat et al., 2022; Kaye et al., 2023; Katip et al., 2024), including 2832 patients, examined the microbiological eradication rate after treatment. There was a significant difference in microbiological eradication rate between the combination therapy group and the monotherapy group (RR 1.07, 95% CI 1.00–1.13, *I²* = 29%, *p* = 0.04) (**Figure 10**). Egger’s test suggested a low level of publication bias (*p* = 0.2527). The subgroup analyses of the observational studies, the RCTs, and the mixed-infection studies revealed no difference, whereas the significant difference was concentrated in the subgroup of CRAB infectious studies (**Supplementary file, Figure S5**).

#### 3.5.5 Nephrotoxicity and neurotoxicity

**Figure 11:**
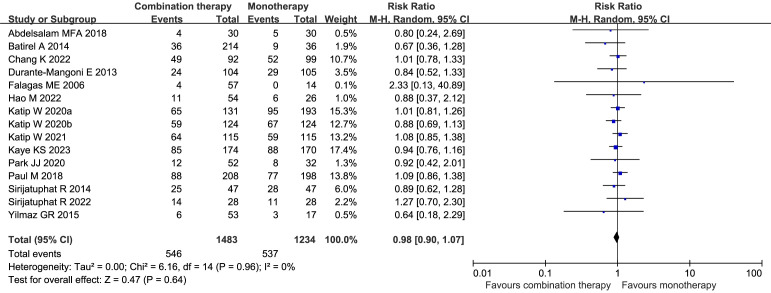
Forest plot of nephrotoxicity between COL combination therapy and monotherapy. Katip W2020a and Katip W2020b denote two independent studies published by the Katip W research team in 2020.

Nephrotoxicity was reported in fifteen studies (2717 patients) (Falagas et al., 2006; Durante-Mangoni et al., 2013; Batirel et al., 2014; Sirijatuphat and Thamlikitkul, 2014; Yilmaz et al., 2015; Abdelsalam et al., 2018; Paul et al., 2018; Katip and Uitrakul, 2020; Katip et al., 2020; Park et al., 2020; Katip and Oberdorfer, 2021; Chang et al., 2022; Hao et al., 2022; Sirijatuphat et al., 2022; Kaye et al., 2023), and overall observed nephrotoxicity did not differ significantly (RR 0.98, 95% CI 0.90–1.07, *I²* = 0%, *p* = 0.64) (**Figure 11**). Egger’s test revealed low publication bias (*p* = 0.3884). Subgroup analyses showed no difference between the observational studies and the RCTs (**Supplementary file, Figure S6**).

**Figure 12:**
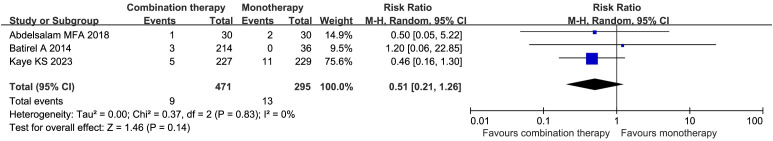
Forest plot of neurotoxicity between COL combination therapy and monotherapy.

Similarly, three studies (766 patients) also revealed no significant difference (RR 0.51, 95% CI 0.21–1.26, *I²* = 0, *p* = 0.14) in neurotoxicity incidence (**Figure 12**) (Batirel et al., 2014; Abdelsalam et al., 2018; Kaye et al., 2023). Egger’s test showed a low risk of publication bias (*p* = 0.4083). Additionally, subgroup analyses of RCTs reported no significant differences (**Supplementary file**; **Figures S7**).

For all secondary outcomes, sensitivity analysis via the leave-one-out method confirmed the robustness of the findings. Moreover, the funnel plots (**Supplementary file**; **Figures S8-S14**) showed no evidence of publication bias.

### 3.6 GRADE certainty of evidence

Evidence quality for 28-day mortality and microbiological clearance rate was rated as moderate, primarily because the included studies carried a moderate risk of bias. Evidence quality for in-hospital mortality, clinical improvement rate, total hospital stay duration, ICU stay duration, nephrotoxicity, and neurotoxicity was rated as low, mainly due to a high risk of bias and imprecision in the included studies.

## 4 Discussion

This systematic review compared the clinical outcomes between COL combination therapy and monotherapy for CR-GNB infections. The results showed that COL combination therapy was associated with higher microbial eradication rates in CRAB infections. However, no statistically significant differences in 28-day all-cause mortality, clinical improvement rates, length of stay, nephrotoxicity incidence, or neurotoxicity incidence were detected between the two groups. Subgroup analyses of primary outcomes stratified by study design (RCTs and observational studies), study setting (multicenter vs. single-center), pathogen subtype (CRAB-infected patients only), antibiotic regimen (COL + meropenem, COL+ tigecycline, COL + rifampicin), and baseline severity (critically ill and stable patients) also supported this finding.

The primary outcomes of this study align with findings from nine previous systematic reviews (Chen et al., 2015; Zusman et al., 2017; Cheng et al., 2018; Kengkla et al., 2018; Vardakas et al., 2018; Schmid et al., 2019; Wang et al., 2019; Samal et al., 2021; Huang et al., 2022), including four that focused exclusively on CRAB infections (Chen et al., 2015; Kengkla et al., 2018; Wang et al., 2019; Huang et al., 2022). These findings confirmed that COL combination therapy did not improve survival outcomes. For secondary outcomes, the effect estimates for clinical improvement rates in this study also showed the same direction of effect as those reported in three previous systematic reviews (Gu et al., 2014; Schmid et al., 2019; Huang et al., 2022). With respect to microbiological endpoints, systematic reviews focusing exclusively on CRAB infections demonstrated significantly higher microbiological eradication rates with COL combination therapy than with monotherapy (Chen et al., 2015; Wang et al., 2019; Huang et al., 2022). However, previous studies revealed no significant differences between the two groups when mixed infections involving CRAB, Carbapenem-resistant *Pseudomonas aeruginosa* (CRPA), and other multidrug-resistant pathogens were analyzed (Gu et al., 2014). This is consistent with the subgroup analysis results of our study. These data indicate that COL combination therapy has a microbiological advantage for CRAB (RR = 1.12; 95% CI 1.03–1.21), but no advantage has been found for mixed infections. This may be influenced by differences in pathogens. The microbiological advantage of COL combination therapy for CRAB suggests synergistic effects between COL and other antibiotics. Research indicates synergistic bactericidal effects when COL is combined with sulbactam, ampicillin/sulbactam, cefoperazone/sulbactam, imipenem, meropenem, amikacin, tigecycline, fosfomycin, or rifampicin (Ardebili et al., 2023; Wang et al., 2024). This synergism may potentially enhance antibacterial activity and improve microbiological eradication rates in CRAB infections. Nevertheless, the microbial advantage of combination therapy did not translate into survival benefits. This may be attributed to higher baseline disease severity/confounding, greater comorbidity burden among patients receiving combination therapy, and limited lung tissue penetration of COL (Rodvold et al., 2011; Rottbøll and Friis, 2016). Inappropriate use of combination therapy may increase selective pressure and toxicity.

Additionally, this study evaluated the incidence of LOS, nephrotoxicity, and neurotoxicity between the two therapeutic strategies. The results revealed no statistically significant differences in LOS, nephrotoxicity, or neurotoxicity rates between COL combination therapy and monotherapy. However, the confidence interval for the LOS outcome was wide; thus, this finding must be interpreted cautiously, most likely because few studies contributed to the LOS analysis. Nevertheless, leave-one-out sensitivity analyses indicated that the direction of the effect remained unchanged after the sequential exclusion of any single study. These findings suggest that combination therapy may neither shorten the duration of hospital stay nor confer additional drug-related toxicity. Based on these outcomes, the study aligns with guidelines from the Infectious Diseases Society of America and the European Society of Clinical Microbiology and Infectious Diseases, which recommend COL combination therapy for CR-GNB infections, particularly CRAB infections (Paul et al., 2022; Tamma et al., 2024). These findings provide a reference for the management of antimicrobial agents, particularly in low- and middle-income countries. Previous studies have indicated that COL are extensively utilized in these regions, potentially impacting the resistance patterns of CR-GNB (Iskandar et al., 2021; Umair et al., 2023). However, it must be noted that clinical practice may be more complex due to differences in healthcare resources, patient characteristics, and regional resistance profiles. Consequently, clinicians should adapt these research outcomes to their specific contexts while considering both the potential benefits and risks of combination therapy.

However, the heterogeneity of the study results should also be taken into consideration when interpreting these findings. The I² for the primary outcome (28-day all-cause mortality) in this meta-analysis was 25%, while the I² for secondary outcomes ranged from 0% to 48%, indicating the presence of statistical heterogeneity across the results. We attribute the heterogeneity of these results to several factors. First, most of the included studies have a high risk of bias. Second, there was substantial variability in the combination therapy regimens in our study, which not only included COL combined with different antibiotics such as meropenem, imipenem, amikacin, gentamicin, and tigecycline in dual therapy but also involved triple therapy with COL combined with meropenem and tigecycline. Different combinations of medications may have varying therapeutic effects. Third, there were differences in the types of pathogens included in this article, which encompassed pure or mixed infections of CRAB, CRE, and CRPA. The distinct resistance profiles and virulence factors of these pathogens may have led to varying treatment responses. Fourth, there were differences in study design and setting in our research. These factors contributed to the occurrence of heterogeneity, thereby limiting the generalizability of the study results and affecting the judgment of the superior intervention. Moreover, recent studies have indicated that there are significant regional differences in the resistance rates and patterns of CR-GNB. For instance, the highest COL resistance rates for CRAB have been reported in Western Europe and South America (Bostanghadiri et al., 2024), while higher resistance rates for CRE have been noted in South Africa and Nigeria (Gashaw et al., 2025). Additionally, the resistance rates to the antibiotics used in combination therapy also vary by pathogen and region. For example, the resistance rate of CRAB to tigecycline is as high as 66% in Israel, whereas the resistance rate of CRE to tigecycline can reach 11% in Pakistan (Yaghoubi et al., 2022). These regional differences in CR-GNB resistance rates and patterns, which further complicate treatment choices and impact the efficacy of COL in different regions, can also lead to heterogeneity. To mitigate the impact of between-study heterogeneity on the results, we conducted subgroup analyses based on study design, study setting, pathogen type, antibiotic regimen, and baseline severity. These analyses aimed to assess the robustness of the study findings.

Compared with previous systematic reviews, this study included a larger number of studies and excluded case series with a high risk of bias. However, several limitations remain. First, the search was restricted to three major English-language databases and included a limited number of studies, which may introduce publication bias and compromise the validity of our interpretations. Second, we included only eight RCTs, with the remainder being observational studies. Although nearly all observational studies reported comparable baseline characteristics between groups, inherent selection bias and confounding factors due to the nonrandomized design could not be fully eliminated. Third, significant heterogeneity existed across studies in terms of antibiotic combination regimens and pathogen types, which may have influenced the pooled results. Therefore, although the most recent studies were included and both publication-bias assessment and sensitivity analyses were performed, the limited number of trials and the observed heterogeneity warrant cautious interpretation of the findings. Besides, while this study demonstrated superior microbiological eradication rates with combination therapy, data on the development of COL resistance during treatment were not reported. This gap arises because most included studies did not document COL resistance patterns, precluding further exploration of whether combination therapy mitigates pathogen mutation risk or exerts selective pressure on ICU flora.

In summary, we found that, compared with monotherapy, COL combination therapy may demonstrate superior microbiological eradication rates in CRAB infections while not increasing the risk of nephrotoxicity or neurotoxicity. However, no statistically significant differences were observed between the two strategies in 28-day all-cause mortality, clinical improvement rates, and LOS. Despite the certainty of evidence being moderate for microbiological eradication and 28-day all-cause mortality, and low for all other outcomes, we cautiously suggest that colistin-based combination therapy be considered for CR-GNB infections—especially those caused by CRAB—because it may improve microbiological eradication, although the magnitude of benefit is likely to vary among individual patients. Additionally, it is important to note that, as most available evidence is observational, residual selection bias cannot be excluded. More high-quality randomized trials—with concurrent monitoring for emergent COL resistance—are needed to better define the role of combination therapy in CR-GNB infections.

## Data Availability

The original contributions presented in the study are included in the article/**Supplementary Material**. Further inquiries can be directed to the corresponding author.
