# Supplementary material for: Colistin-based combination therapy versus monotherapy for carbapenem-resistant gram-negative bacterial infections: a systematic review and meta-analysis
Source: Front Cell Infect Microbiol. 2026 Jan 12;15:1729919. doi: 10.3389/fcimb.2025.1729919 (PMC12832914; doi:10.3389/fcimb.2025.1729919)
Supplement: Supplementary file 1 [file DataSheet1.pdf]

Supplementary Material

1 Supplementary table S1: Search strategies

Search engine	Terms
Pubmed (1082)	((((((((((Carbapenem-Resistant[MeSH Terms]) OR (Carbapenemase-Producing[Title/Abstract])) OR (Carbapenem-Resistant[Title/Abstract])) OR (carbapenem resistant[Title/Abstract])) OR (carbapenem nonsusceptible[Title/Abstract])) OR (Multidrug resistant Gram negative bacteria[Title/Abstract])) OR (Multidrug-resistant Gram-negative bacteria[Title/Abstract])) OR (Extensively Drug Resistant Gram-negative Bacilli[Title/Abstract])) OR (XDR-GNB[Title/Abstract])) OR (MDR-GNB[Title/Abstract])) AND ((((((((Colistin[MeSH Terms]) OR (Colimycin[Title/Abstract])) OR (Colisticin[Title/Abstract])) OR (Polymyxin E[Title/Abstract])) OR (Colistin Sulfate[Title/Abstract])) OR (Sulfate, Colistin[Title/Abstract])) OR (Totazina[Title/Abstract])) OR (Coly-Mycin[Title/Abstract]))
Embase （4759）	#1 colistin:ti,ab,kw OR colimycin:ti,ab,kw OR colisticin:ti,ab,kw OR 'polymyxin e': ti,ab,kw OR 'colistin sulfate': ti,ab,kw OR 'sulfate colistin': ti,ab,kw OR totazina:ti,ab,kw OR 'coly mycin':ti,ab,kw<br><br>#2 'carbapenemase producing': ti, ab,kw OR 'carbapenem resistant': ti, ab,kw OR 'carbapenem nonsusceptible': ti, ab,kw OR 'multidrug resistant gram-negative bacteria': ti, ab,kw OR mdr: ti, ab,kw OR 'multidrug-resistant gram-negative bacteria': ti, ab,kw OR 'extensively drug-resistant gram-negative bacilli': ti, ab,kw OR 'xdr gnb': ti, ab,kw<br><br>#3 #1 AND #2

(Continued)

Search engine	Terms
Cochrane Library (159)	#1 MeSH descriptor: [Carbapenem-Resistant Enterobacteriaceae] explode all trees<br><br>#2 (Extensively Drug-Resistant Gram-negative Bacilli): ti, ab,kw OR (XDR-GNB): ti, ab,kw AND (MDR-GNB): ti, ab,kw (Word variations have been searched)<br><br>#3 (Carbapenem-Resistant): ti, ab,kw OR (Carbapenemase-Producing): ti, ab,kw OR (carbapenem-resistant): ti, ab,kw OR (carbapenem nonsusceptible): ti, ab,kw OR (Multidrug-resistant Gram-negative bacteria): ti, ab,kw (Word variations have been searched) 660<br><br>#4 MeSH descriptor: [Colistin] explode all trees<br><br>#5 (Colistin): ti,ab,kw OR ("colisticin"): ti,ab,kw OR (Colimycin): ti,ab,kw OR (Polymyxin E): ti,ab,kw OR ("colistin sulfate"): ti,ab,kw (Word variations have been searched) 664<br><br>#6 (Totazina): ti, ab,kw OR ("Coly Mycin"): ti, ab,kw (Word variations have been searched)<br><br>#7 #1 OR #2 OR #3<br><br>#8 #4 OR #5 OR #6<br><br>#9 #7 AND #8

(Continued)

Supplementary table S2: Characteristics of disease severity, comorbidity, and infection site in the colistin combination versus monotherapy for the included studies.

Study	The severity of the illness			Charlson comorbidity index (median, IQR)			Primary infection site (%)	Site of infection, n							
	Total	Mono	Combine	Total	Mono	Combine		Blood		Respiratory		IAI		UTI	
								Mono	Combine	Mono	Combine	Mono	Combine	Mono	Combine
Abdelsalam MFA 2018	NR	18.87 ± 5.463[2]	18.13 ± 4.023[2]	NR	NR	NR	PNA (100%), UTI (17%), ABI (11.7%), SSI (3%), SSTI (2%); CRBSI (15%), Bacteremia (46.67%)	11	17	30	30	2	5	7	3
Amat T 2018	23 ± 7[2]	23 ± 7[2]	24 ± 7[2]	2.7 ± 2	2.6 ± 1.8	2.7 ± 2.4	PNA (64%), SSSI, CNS, SSI,	63	34	50	26	NR	NR	NR	NR
Aydemir H 2013	19·1 ± 6·0[2]	18·0 ± 4·9[2]	20·1 ± 6·8[2]	NR	NR	NR	Bacteremia (18·6%)	3	5	NR	NR	NR	NR	NR	NR
Batirel A 2014	NR	17.9 ± 7.1[2]	18.6 ± 6.9[2]	NRs	3.5±2.2	3.3±2.2	NR	NR	NR	NR	NR	NR	NR	NR	NR
Chang K 2022	NR	17.00 ± 11.00[2]	18.00 ± 10.00$_2$	NR	2.00 ± 2.00	2.00 ± 2.00	NR	NR	NR	NR	NR	NR	NR	NR	NR
Durante-Mangoni E 2013	NR	39.0 ±11.1[4]	40.8 ±10.8[4]	NR	NR	NR	PNA (77.5%), Bacteremia (20.1%), IAI (2.4%)	21	21	81	81	3	2	NR	NR

(Continued)

Supplementary table S2: Characteristics of disease severity, comorbidity, and infection site in the colistin combination versus monotherapy for the included studies.

Study	The severity of the illness			Charlson comorbidity index (median, IQR)			Primary infection site (%)	Site of infection, n							
	Total	Mono	Combine	Total	Mono	Combine		Blood		Respiratory		IAI		UTI	
								Mono	Combine	Mono	Combine	Mono	Combine	Mono	Combine
Falagas ME 2006	NR	14.3 ± 7.4[2]	15.4 ± 6.6[2]	NR	NR	NR	PNA (40.84%), UTI (9.86%), IAI (9.86%), SSTI (4.23%), bacteremia (23.94%), SD (2.82%)	1	16	6	23	2	5	4	3
Ghafur A 2016	NR	20.4±5.3[2]	24.1±7.1[2]	NR	5.4±3.2	5.2±2.8	NR	NR	NR	NRR	NR	NR	NR	NR	NR
Ghafur A 2017	NR	NR	NR	NR	NR	NR	PNA (71.16%), UTI (11.11%), CNS (1.31%), IAI (0.65%)	NR	NR	454	70	0	1	10	7
Hao M 2022	NR	NR	NR	NR	NR	NR	PNA (67.5%), bacteremia (15%), UTI (5%), CNS (3.75%), IAI (6.25%)	6	6	17	37	0	5	3	1
Kalin G 2014	NR	22 (14–36)[2]	27 (18–35)[2]	NR	NR	NR	NR	NR	NR	NR	NR	NR	NR	NR	NR

(Continued)

Supplementary table S2: **Characteristics of disease severity, comorbidity, and infection site in the colistin combination versus monotherapy for the included studies.**

Study	The severity of the illness			Charlson comorbidity index (median, IQR)			Primary infection site (%)	Site of infection, n							
	Total	Mono	Combine	Total	Mono	Combine		Blood		Respiratory		IAI		UTI	
								Mono	Combine	Mono	Combine	Mono	Combine	Mono	Combine
Katip W 2020-8	NR	NR	NR	NR	1.74 + 0.13	2.38 + 0.16	PNA (34.26%), bacteremia (35.49%), UTI (15.12%)	61	54	59	52	NR	NR	37	12
Katip W 2020-9	NR	17.08 ± 4.47[2]	17.32 ± 4.53[2]	NR	2 (1–3.5)	2 (1–4)	PNA (0.81%), bacteremia (88.71%), UTI (6.85%)	0	2	109	111	NR	NR	7	10
Katip W 2021	NR	12.53 ± 0.49 [2]	11.92 ± 0.59 [2]	NR	2 (0–3)	2 (1–4)（IQR）	PNA (0.43%), bacteremia (88.70%), UTI (9.57%)	1	0	101	103	NR	NR	12	10
Katip W 2024	NR	8.22+ 6.42 [2]	9.93+ 6.66[2]	NR	NRR	NR	PNA (7.27%), bacteremia (26.82%), UTI (53.18%)	4	12	13	46	NR	NR	38	79
Kaye KS 2023	IQR	22 (17–26)[2]	21 (17–26)[2]	NR	5 (4–7)	5 (4–7)	PNA (29.55%), bacteremia (70.45%)	61	64	152	146	NR	NR	NR	NR
Makris D 2018	NR	14.5±3.1[2]	16.5±4.7[2]	NR	NR	NR	PNA (100%), bacteremia (46.15%)	9	9	19	20	NR	NR	NR	NR

(Continued)

Supplementary table S2: Characteristics of disease severity, comorbidity, and infection site in the colistin combination versus monotherapy for the included studies.

Study	The severity of the illness			Charlson comorbidity index (median, IQR)			Primary infection site (%)	Site of infection, n							
	Total	Mono	Combine	Total	Mono	Combine		Blood		Respiratory		IAI		UTI	
								Mono	Combine	Mono	Combine	Mono	Combine	Mono	Combine
Park JJ 2020	NR	12.4 ± 6.16[2]	12.1 ± 5.72[2]	Range	0 （0-6）	0 （0-5）	SSTI（100%）	NR	NR	NR	NR	NR	NR	NR	NR
Park SY 2019	NR	4.05 ± 2.51[1]	3.93 ± 2.30[1]	NR	3.20 ± 1.77	3.32 ± 1.44	CRBSI（38.03%, PNA (43.66%), Bacteremia (1.41%), UTI (1.41%), IAI (4.23%)	1	0	15	16	2	1	0	1
Paul M 2018	IQR	6 (3–8)[3]	5 (4–8)[3]	NR	2 (0–3)	2 (0–4)	Bacteremia (42.61%), PNA (50.99%), UTI (6.40%)	76	97	108	99	NR	NR	14	12
Rigatto MH 2015	NR	19.5 ± 6.4[2]	20.8 ± 7.4[2]	NR IQR	2 (1–3.75)	2 (1–5)	PNA (81.19%), Bacteremia (18.81%), UTI (3.96%), IAI (5.94%)	14	5	57	25	3	3	3	1
Shi H 2019	NR	＞24 score[2]：27	＞24 score[2]：50	NR	NR	NR	PNA (100%)	NR	NR	77	83	NR	NR	NR	NR
Sirijatuphat R 2014	NR	21.9 ± 7.9[2]	23.0 ± 6.4[2]	NR	NR	NR	PNA (76.60%), bacteremia (5.32%), UTI (5.32%), IAI (6.38%)	3	2	35	37	4	2	2	3

(Continued)

Supplementary table S2: Characteristics of disease severity, comorbidity, and infection site in the colistin combination versus monotherapy for the included studies.

Study	The severity of the illness			Charlson comorbidity index (median, IQR)			Primary infection site (%)	Site of infection, n							
	Total	Mono	Combine	Total	Mono	Combine		Blood		Respiratory		IAI		UTI	
								Mono	Combine	Mono	Combine	Mono	Combine	Mono	Combine
Sirijatuphat R 2022	18.1 ± 5.2[2]	18.0 ± 4.9[2]	18.1 ± 5.6[2]	NR	NR	NR	SSTI (1.79%), PNA (58.93%), bacteremia (21.43%), UTI (5.36%), IAI (3.57%)	6	6	20	13	0	2	2	1
Yilmaz GR 2015	NR	43.8 ± 12.1[4]	50.8 ± 11.73[4, 5]	NR	NR	NR	PNA (100%)	NR	NR	17	53	NR	NR	NR	NR
Parchem NL 2016	NR	24±7.41[2]	25±8.89[2]	NR	3±2.96	4±1.48	PNA (100%)	NR	NR	31	25	NR	NR	NR	NR

Abbreviations: PNA, pneumonia; SSTI, skin and soft tissue infection; UTI, urinary tract infection; ABI, abdominal infection; CNS, central venous system; BTI, biliary tract infection; CRBSI, catheter-related bloodstream infection; LRTI, lower respiratory tract infection; CVC, central venous catheter; PJI, periprosthetic joint infection; SSI, surgical site infection; IAI, intra-abdominal infection; SD, spondylodiscitis; NR, no report.

[1]Pitt bacteremia score; [2]APACHE-II Score; [3]SOFA score; [4]SAPS II score; [5]The results mixed with the data from other study groups.

3 Supplementary table S3: GRADE Certainty of Evidence

Outcome	Quality of Evidence	Risk of Bias	Imprecision	Inconsistency	Indirectness	Publication Bias
28-day mortality	Moderate	Moderate	None	Low ($I^2 = 25\%$)	None	None
In-hospital mortality	Low	High	Moderate	Moderate ($I^2 = 40\%$)	None	None
Microbiological clearance rate	Moderate	Moderate	None	Low ($I^2 = 19\%$)	None	None
Clinical improvement rate	Low	High	None	Moderate ($I^2 = 48\%$)	None	None
Total hospital stay duration	Low	High	Present (Wide confidence intervals)	Moderate ($I^2 = 37\%$)	None	None
ICU stay duration	Low	High	None	Low ($I^2 = 19\%$)	None	None
Nephrotoxicity	Low	High	Present (Wide confidence intervals)	None ($I^2 = 0\%$)	None	None
Neurotoxicity	Low	High	Present (Wide confidence intervals)	None ($I^2 = 0\%$)	None	None

4 Supplementary Figures:

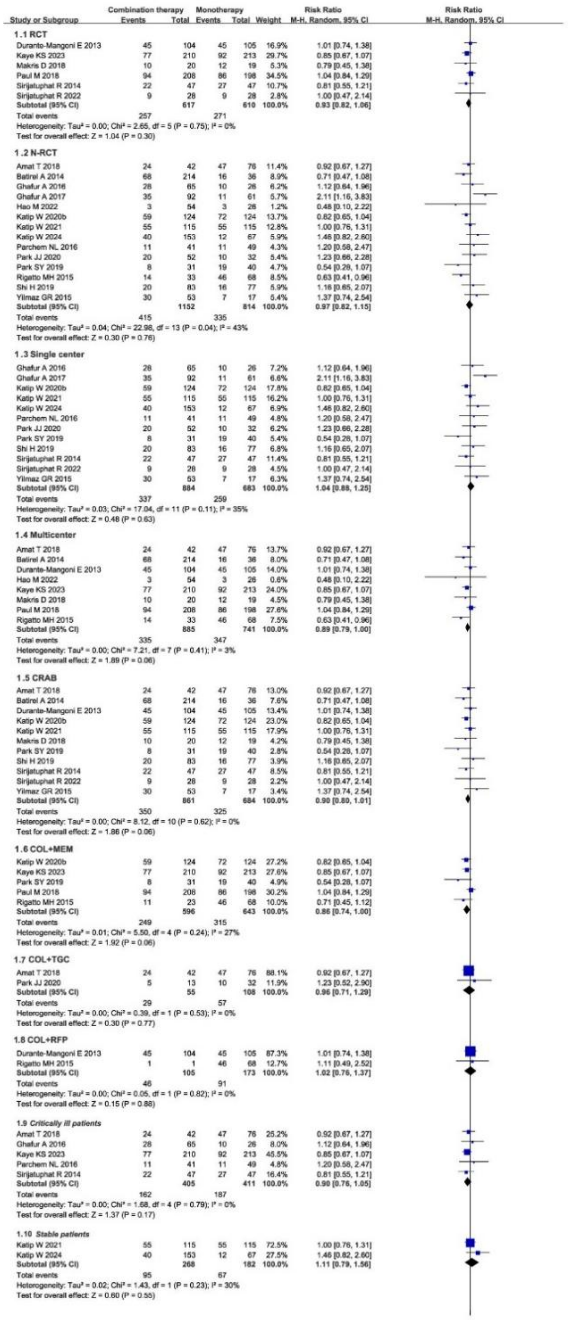

Supplementary Figure S1. Subgroup analysis of 28-day mortality of the colistin (COL) combination therapy versus monotherapy. The results were stratified by study design (RCTs and observational studies), study setting (multicenter vs. single-center), pathogen subtype (CRAB-infected patients only) and antibiotic regimen (COL+MEM, COL+TGC, COL+RFP). MEM, meropenem; TGC, tigecycline; RFP, rifampicin

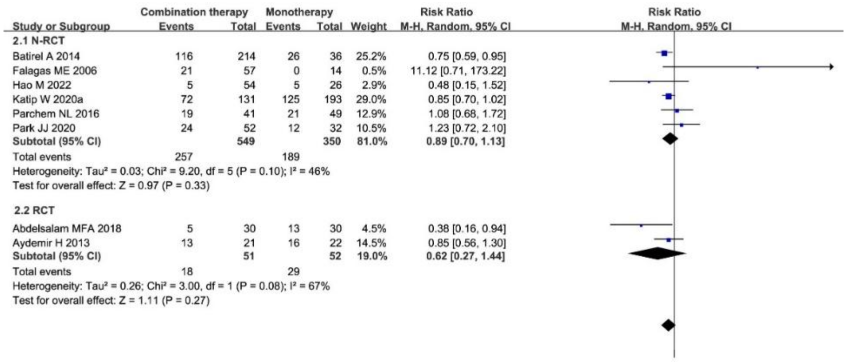

Supplementary Figure S2. Subgroup analysis of in-hospital mortality rates of the COL combination therapy versus monotherapy. The results were stratified by study design (RCTs and observational studies)

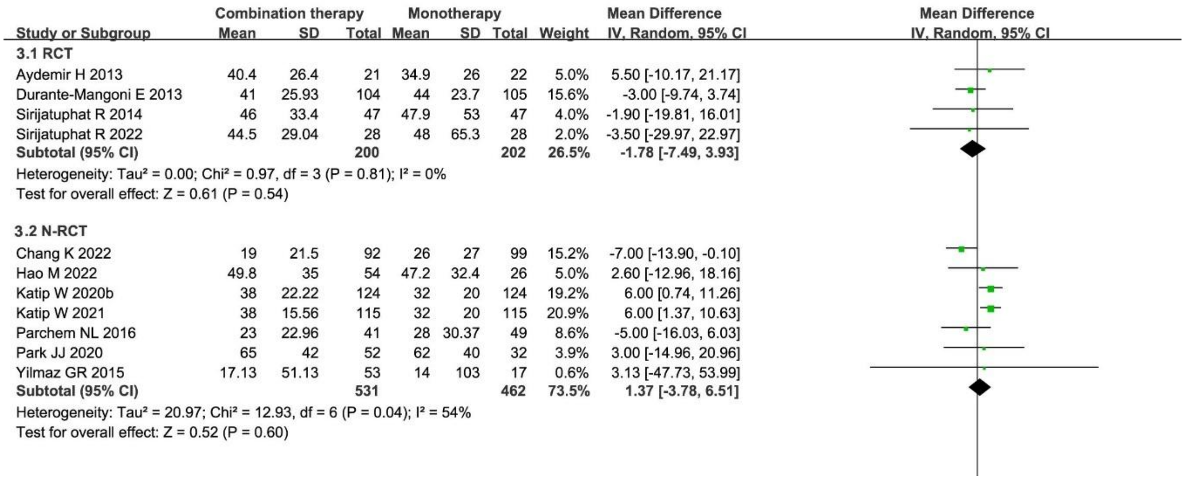

Supplementary Figure S3. Subgroup analysis of total hospital LOS of the COL combination therapy versus monotherapy. The results were stratified by study design (RCTs and observational studies). LOS, length of stay.

	Combination therapy		Monotherapy			Risk Ratio
**Study or Subgroup**	**Events**	**Total**	**Events**	**Total**	**Weight**	**M-H, Random, 95% CI**
**4.1 RCT**						
Aydemir H 2013	11	21	9	22	0.4%	1.28 [0.67, 2.45]
Kaye KS 2023	80	190	65	184	1.8%	1.19 [0.92, 1.54]
Makris D 2018	10	20	10	19	0.5%	0.95 [0.52, 1.75]
Paul M 2018	56	208	42	198	1.2%	1.27 [0.89, 1.80]
Sirijatuphat R 2014	28	47	26	47	1.2%	1.08 [0.76, 1.53]
Sirijatuphat R 2022	22	28	21	28	1.6%	1.05 [0.79, 1.40]
**Subtotal (95% CI)**		**514**		**498**	**6.7%**	**1.14 [0.98, 1.31]**
Total events	207		173			
Heterogeneity: Tau² = 0.00; Chi² = 1.50, df = 5 (P = 0.91); I² = 0%						
Test for overall effect: Z = 1.75 (P = 0.08)						
**4.2 N-RCT**						
Batirel A 2014	167	214	27	36	2.3%	1.04 [0.85, 1.27]
Chang K 2022	25	92	41	99	0.9%	0.66 [0.44, 0.99]
Falagas ME 2006	39	57	12	14	1.6%	0.80 [0.60, 1.05]
Ghafur A 2017	57	92	50	61	2.4%	0.76 [0.62, 0.92]
Hao M 2022	51	54	19	26	1.9%	1.29 [1.01, 1.65]
Kalin G 2014	14	35	14	47	0.5%	1.34 [0.74, 2.44]
Katip W 2020a	80	131	130	193	2.7%	0.91 [0.77, 1.07]
Katip W 2020b	74	124	65	124	2.1%	1.14 [0.91, 1.42]
Katip W 2021	67	115	65	115	2.1%	1.03 [0.82, 1.29]
Katip W 2024	110	153	57	67	3.1%	0.85 [0.73, 0.97]
Parchem NL 2016	23	41	31	49	1.2%	0.89 [0.63, 1.25]
Park SY 2019	19	31	16	40	0.7%	1.53 [0.96, 2.46]
Shi H 2019	47	83	46	77	1.8%	0.95 [0.73, 1.23]
Yilmaz GR 2015	42	53	13	17	1.5%	1.04 [0.77, 1.40]
**Subtotal (95% CI)**		**1275**		**965**	**25.0%**	**0.96 [0.87, 1.06]**
Total events	815		586			
Heterogeneity: Tau² = 0.02; Chi² = 28.61, df = 13 (P = 0.007); I² = 55%						
Test for overall effect: Z = 0.73 (P = 0.46)						
**4.3 Single center**						
Aydemir H 2013	11	21	9	22	0.4%	1.28 [0.67, 2.45]
Falagas ME 2006	39	57	12	14	1.6%	0.80 [0.60, 1.05]
Ghafur A 2017	57	92	50	61	2.4%	0.76 [0.62, 0.92]
Kalin G 2014	14	35	14	47	0.5%	1.34 [0.74, 2.44]
Katip W 2020a	80	131	130	193	2.7%	0.91 [0.77, 1.07]
Katip W 2020b	74	124	65	124	2.1%	1.14 [0.91, 1.42]
Katip W 2021	67	115	65	115	2.1%	1.03 [0.82, 1.29]
Katip W 2024	110	153	57	67	3.1%	0.85 [0.73, 0.97]
Parchem NL 2016	23	41	31	49	1.2%	0.89 [0.63, 1.25]
Park SY 2019	19	31	16	40	0.7%	1.53 [0.96, 2.46]
Shi H 2019	47	83	46	77	1.8%	0.95 [0.73, 1.23]
Sirijatuphat R 2014	28	47	26	47	1.2%	1.08 [0.76, 1.53]
Sirijatuphat R 2022	22	28	21	28	1.6%	1.05 [0.79, 1.40]
Yilmaz GR 2015	42	53	13	17	1.5%	1.04 [0.77, 1.40]
**Subtotal (95% CI)**		**1011**		**901**	**23.0%**	**0.96 [0.88, 1.05]**
Total events	633		555			
Heterogeneity: Tau² = 0.01; Chi² = 21.03, df = 13 (P = 0.07); I² = 38%						
Test for overall effect: Z = 0.92 (P = 0.36)						
**4.4 Multicenter**						
Batirel A 2014	167	214	27	36	2.3%	1.04 [0.85, 1.27]
Chang K 2022	25	92	41	99	0.9%	0.66 [0.44, 0.99]
Hao M 2022	51	54	19	26	1.9%	1.29 [1.01, 1.65]
Kaye KS 2023	80	190	65	184	1.8%	1.19 [0.92, 1.54]
Makris D 2018	10	20	10	19	0.5%	0.95 [0.52, 1.75]
Paul M 2018	56	208	42	198	1.2%	1.27 [0.89, 1.80]
**Subtotal (95% CI)**		**778**		**562**	**8.7%**	**1.09 [0.91, 1.29]**
Total events	389		204			
Heterogeneity: Tau² = 0.02; Chi² = 9.47, df = 5 (P = 0.09); I² = 47%						
Test for overall effect: Z = 0.93 (P = 0.35)						
**4.5 CRAB**						
Aydemir H 2013	11	21	9	22	0.4%	1.28 [0.67, 2.45]
Batirel A 2014	167	214	27	36	2.3%	1.04 [0.85, 1.27]
Katip W 2020a	80	131	130	193	2.7%	0.91 [0.77, 1.07]
Katip W 2020b	74	124	65	124	2.1%	1.14 [0.91, 1.42]
Katip W 2021	67	115	65	115	2.1%	1.03 [0.82, 1.29]
Makris D 2018	10	20	10	19	0.5%	0.95 [0.52, 1.75]
Park SY 2019	19	31	16	40	0.7%	1.53 [0.96, 2.46]
Shi H 2019	47	83	46	77	1.8%	0.95 [0.73, 1.23]
Sirijatuphat R 2014	28	47	26	47	1.2%	1.08 [0.76, 1.53]
Sirijatuphat R 2022	22	28	21	28	1.6%	1.05 [0.79, 1.40]
Yilmaz GR 2015	42	53	13	17	1.5%	1.04 [0.77, 1.40]
**Subtotal (95% CI)**		**867**		**718**	**17.0%**	**1.03 [0.95, 1.11]**
Total events	567		428			
Heterogeneity: Tau² = 0.00; Chi² = 6.70, df = 10 (P = 0.75); I² = 0%						
Test for overall effect: Z = 0.62 (P = 0.54)						
**4.6 COL+MEM**						
Falagas ME 2006	39	57	12	14	1.6%	0.80 [0.60, 1.05]
Katip W 2020a	80	131	130	193	2.7%	0.91 [0.77, 1.07]
Katip W 2020b	74	124	65	124	2.1%	1.14 [0.91, 1.42]
Kaye KS 2023	80	190	65	184	1.8%	1.19 [0.92, 1.54]
Park SY 2019	19	31	16	40	0.7%	1.53 [0.96, 2.46]
Paul M 2018	56	208	42	198	1.2%	1.27 [0.89, 1.80]
**Subtotal (95% CI)**		**741**		**753**	**10.2%**	**1.07 [0.90, 1.27]**
Total events	348		330			
Heterogeneity: Tau² = 0.03; Chi² = 12.66, df = 5 (P = 0.03); I² = 61%						
Test for overall effect: Z = 0.77 (P = 0.44)						
**4.7 Critically ill patients**						
Kaye KS 2023	80	190	65	184	1.8%	1.19 [0.92, 1.54]
Parchem NL 2016	23	41	31	49	1.2%	0.89 [0.63, 1.25]
Sirijatuphat R 2014	28	47	26	47	1.2%	1.08 [0.76, 1.53]
**Subtotal (95% CI)**		**278**		**280**	**4.2%**	**1.07 [0.90, 1.28]**
Total events	131		122			
Heterogeneity: Tau² = 0.00; Chi² = 1.87, df = 2 (P = 0.39); I² = 0%						
Test for overall effect: Z = 0.78 (P = 0.43)						
**4.8 Stable patients**						
Katip W 2021	67	115	65	115	2.1%	1.03 [0.82, 1.29]
Katip W 2024	110	153	57	67	3.1%	0.85 [0.73, 0.97]
**Subtotal (95% CI)**		**268**		**182**	**5.2%**	**0.92 [0.75, 1.13]**
Total events	177		122			
Heterogeneity: Tau² = 0.01; Chi² = 2.56, df = 1 (P = 0.11); I² = 61%						
Test for overall effect: Z = 0.81 (P = 0.42)						

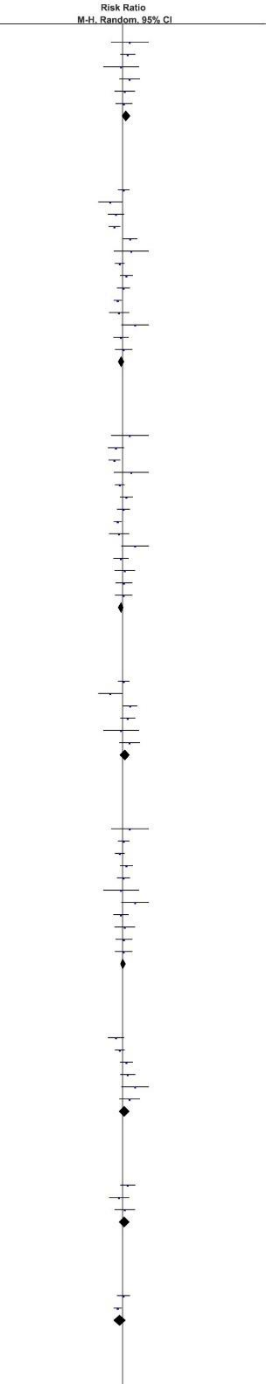

Supplementary Figure S4. Subgroup analysis of clinical improvement rates of the COL combination therapy versus monotherapy. The results were stratified by study design (RCTs and observational studies).

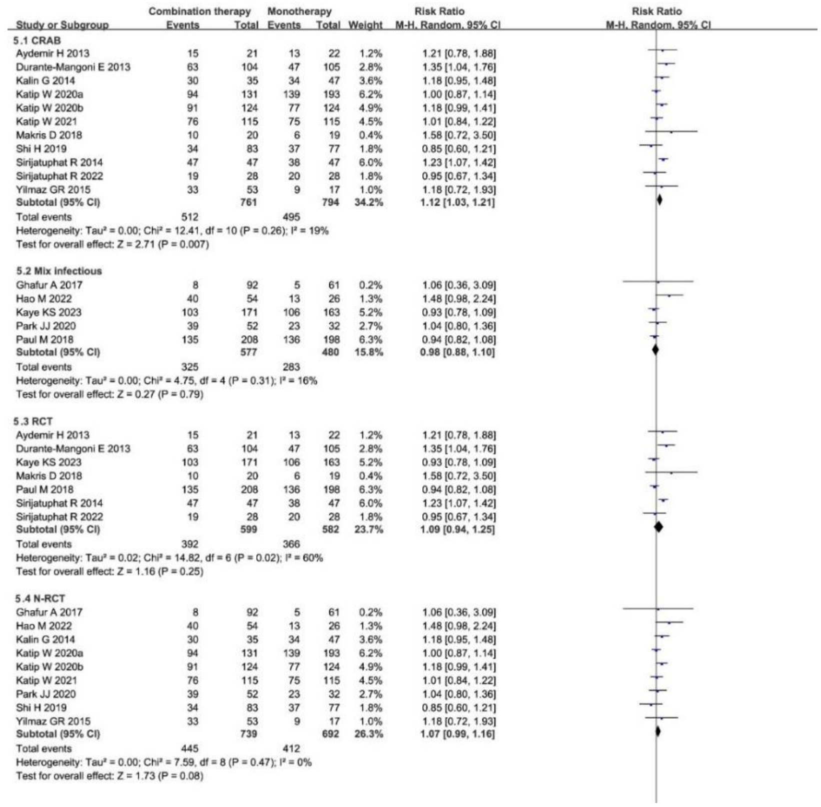

Supplementary Figure S5. Subgroup analysis of microbiological eradication rates of the COL combination therapy versus monotherapy. The results were stratified by study design (RCTs and observational studies) and pathogen subtype (CRAB-infected or mixed pathogen infections).

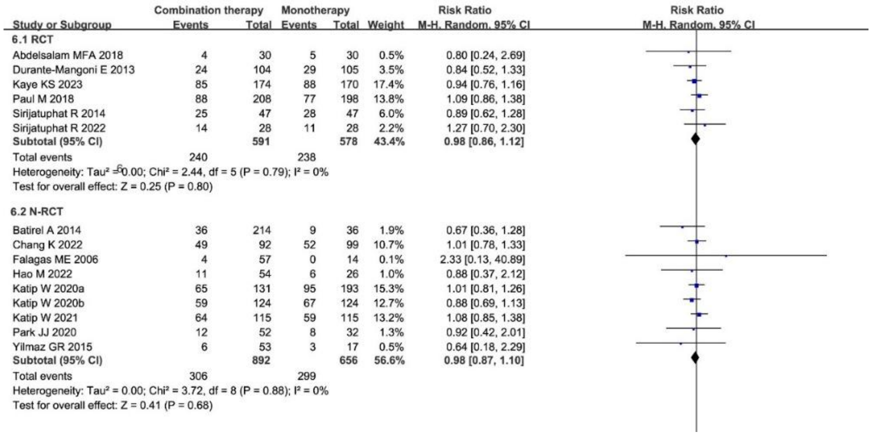

Supplementary Figure S6. Subgroup analysis of nephrotoxicity of the COL combination therapy versus monotherapy. The results were stratified by study design (RCTs and observational studies).

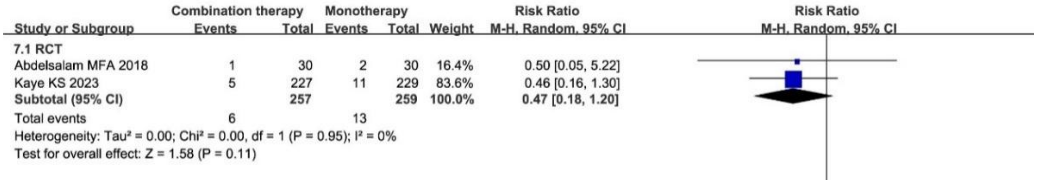

Supplementary Figure S7. Subgroup analysis of neurotoxicity of the COL combination therapy versus monotherapy. The results were stratified by study design (RCTs and observational studies).

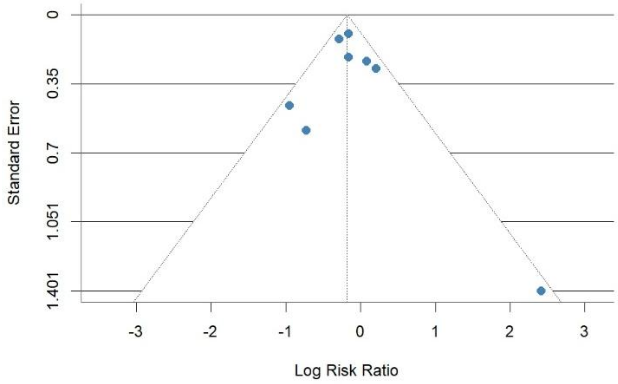

Supplementary Figure S8. Funnel plot of in-hospital mortality rates of the COL combination therapy versus monotherapy.

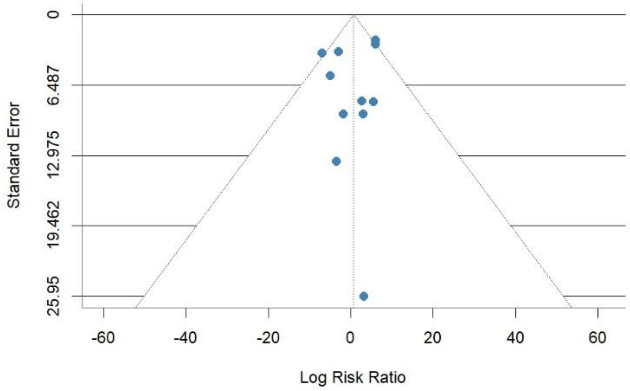

Supplementary Figure S9. Funnel plot of total hospital LOS of the COL combination therapy versus monotherapy. LOS, length of stay.

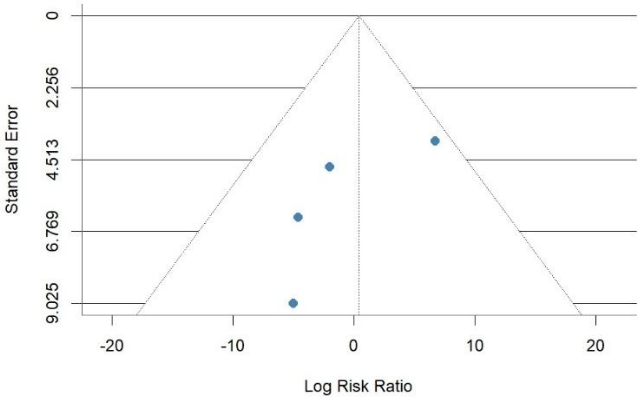

Supplementary Figure S10. Funnel plot of ICU LOS rates of the COL combination therapy versus monotherapy.

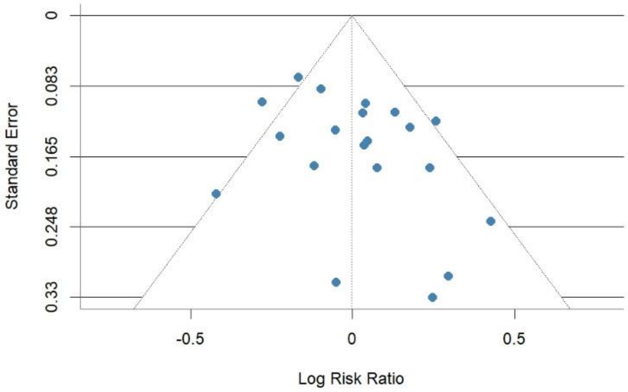

Supplementary Figure S11. Funnel plot of clinical improvement rates of the COL combination therapy versus monotherapy.

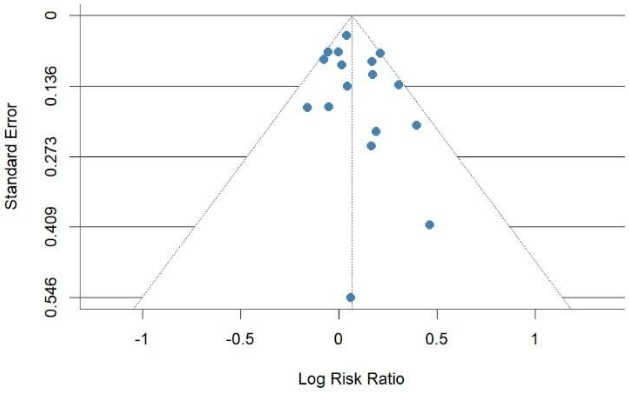

Supplementary Figure S12. Funnel plot of microbiological eradication rates of the COL combination therapy versus monotherapy.

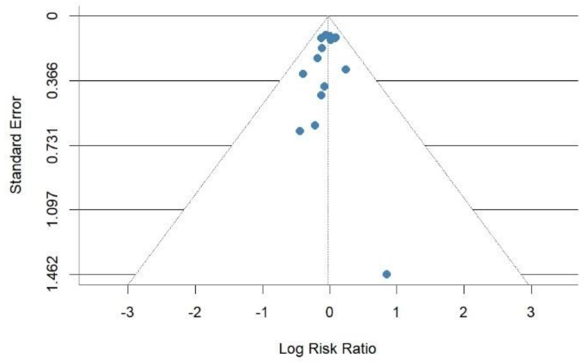

Supplementary Figure S13. Funnel plot of nephrotoxicity of the COL combination therapy versus monotherapy.

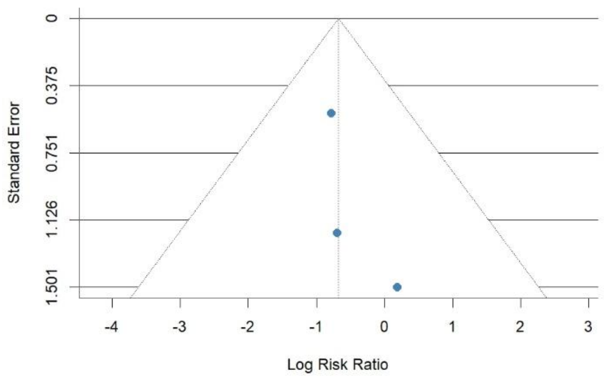

Supplementary Figure S14. Funnel plot of neurotoxicity of the COL combination therapy versus monotherapy.
